# Synthesis and Evaluation of a Selective Fluorogenic Pup Derived Assay Reagent for Dop, a Potential Drug Target in *Mycobacterium tuberculosis*

**DOI:** 10.1002/cbic.201200460

**Published:** 2012-08-24

**Authors:** Remco Merkx, Kristin E Burns, Paul Slobbe, Farid El Oualid, Dris El Atmioui, K Heran Darwin, Huib Ovaa

**Affiliations:** [a] Division of Cell Biology, Netherlands Cancer InstitutePlesmanlaan 121, 1066 CX Amsterdam (The Netherlands); [b] Department of Microbiology, New York University School of Medicine550 First Avenue, MSB 236, New York, NY 10016 (USA)

**Keywords:** deamidase of Pup (Dop), fluorescent probes, protein–protein interactions, Pup-proteasome system (PPS), tuberculosis

Tuberculosis (TB), one of the leading causes of death in the world (http://www.who.int/tb/publications/2011/factsheet_tb_2011.pdf), results from infection with *Mycobacterium tuberculosis* (Mtb). The special properties of the Mtb cell wall combined with its extremely slow dividing time make efficient treatment of TB difficult. Current therapies make use of a combination of antibiotics that are taken daily for many months. Treatment can often be accompanied by severe side effects. Moreover, multidrug-resistant strains of Mtb have developed. Therefore, the need for new TB drugs that inhibit targets that are different from those of currently used drugs is urgent. To minimize side effects these new targets should ideally only be present in the disease-causing bacteria and not in the human host.

Mtb is one of the few bacterial species with a proteasome, a large protein complex that degrades proteins that have been marked for destruction. The recently discovered Pup-proteasome system (PPS), also present in *Actinobacteria* and *Nitrospira*, is essential for the full virulence of Mtb in vivo.^1^] In this pathway, bacterial proteins are post-translationally modified with the small protein prokaryotic ubiquitin-like protein (Pup) to target them for degradation by the proteasome (Scheme 1).^2^] Prior to ligation to target proteins, newly synthesized Pup is activated by deamidase of Pup (Dop).^3^] Dop catalyzes the deamidation of Pup’s C-terminal glutamine to form a glutamate. Attachment of Pup occurs through the newly formed glutamyl side-chain carboxylate to the ε-amino moiety of a lysine residue of the target protein and is catalyzed by proteasome accessory factor A (PafA), the Pup ligase.^4^] The pupylated proteins are then guided to the proteasome through the binding of Pup to mycobacterial proteasome ATPase (Mpa), which unfolds proteins prior to delivery into the proteasome core composed of PrcA and PrcB.^5^] Dop can also function as a depupylase to remove and thereby recycle Pup from substrate proteins prior to proteasomal destruction.^6^] All six proteins are required for a functional Pup–proteasome system (PPS).

**Scheme 1:**
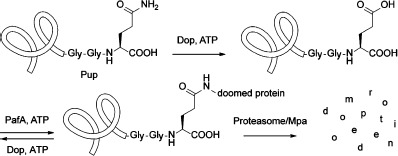
Pupylation pathway of the Pup–proteasome system (PPS) in *Mycobacterium tuberculosis* (Mtb).

Proteasomes are found in all eukaryotes and proteins are generally targeted for proteasomal degradation by post-translational modification with a 76-amino-acid protein called ubiquitin (Ub). The reversible covalent attachment of Ub occurs typically through an isopeptide bond between the C-terminal carboxylate of Ub and the ε-amino moiety of a lysine side chain in the target protein or in Ub itself. The Ub–proteasome system (UPS) is well studied and essential to normal cell function; therefore the enzymes of this pathway are recognized as attractive drug targets for various human diseases.^7^]

Despite the functional homology and analogous mechanism of the PPS and UPS, the similarity of certain proteins between the systems is limited. Most notably, Dop and PafA appear to be unique to bacteria with no known sequence homologues in eukaryotes. Dop and PafA are homologous proteins with similarity to the glutamine synthetase (GS) and γ-glutamyl cysteine ligase (GCS) family of proteins.^8^] PafA catalyzes pupylation in an ATP-dependent reaction, where the gamma carboxylate of Pup’s C-terminal glutamate is phosphorylated prior to attack by a substrate lysine. Interestingly, Dop requires ATP binding but not hydrolysis to catalyse deamidation or depupylation.^3^^,^ ^6a^] Because the PPS is critical for Mtb to cause lethal infections and at least two enzymes in this pathway are unique to bacteria, they provide ideal targets for the development of selective chemotherapies against Mtb. The viability of this approach has recently been demonstrated with the identification of oxathiazol-2-one compounds as selective proteasome inhibitors for a nonreplicating population of Mtb.^1c^^,^ ^9^]

As Dop is required for the full virulence of Mtb,^1f^] the identification of inhibitors against Dop could lead to potential anti-TB lead compounds. However, not much is known about the mechanism of Dop activity and the identification of drug-like inhibitors requires well-designed high-throughput screening (HTS) assay reagents that are currently not available. Presently, a typical Dop assay consists of manual time points and analysis of reaction products by SDS-PAGE or immunoblotting, an inefficient and nonquantitative method to measure activity. Therefore we sought to develop a fluorogenic assay reagent to probe for Dop activity and use in Dop inhibitor screens.

We envisaged a Dop assay reagent that is based on Pup, its natural substrate, and a reporter group that becomes fluorescent after cleavage from Pup. Introduction of the fluorophore 7-amino-4-methylcoumarin (AMC) to the side chain of the C-terminal glutamate of Pup would enable us to measure Dop activity by monitoring the increase in fluorescence over time, representing the hydrolysis of the AMC moiety from Pup. The use of fluorogenic AMC-peptide substrates is a well-established method for the study of proteolytic activity^10^] and a similar reagent, Ub-AMC, has been used extensively to monitor deubiquitinase activity.^11^] In principle, the relatively small size of Pup and its inherently disordered structure make it amenable to chemical peptide synthesis. However, synthesis of such a reagent is not trivial. Pup consists of 64 amino acids and the introduction of AMC through condensation with carboxylic acids often proceeds sluggishly. We previously have reported a high-yielding Fmoc-based linear solid-phase peptide synthesis (SPPS) of Ub reagents that allows for the incorporation of tags and mutations as well as specific C-terminal modifications in a straightforward manner.^12^] Using similar Fmoc SPPS protocols, with the incorporation of dipeptide pseudoproline building blocks at positions 21/22 (Fmoc-Ser-Thr(Ψ^Me,Me^pro)-OH) and 32/33 (Fmoc-Leu-Thr(Ψ^Me,Me^pro)-OH, we synthesized Pup lacking the C-terminal Glu residue (Pup(1–63)), which was confirmed by HPLC-MS analysis. Omission of the dipeptide building blocks led to a less productive synthesis (see Supporting Information). The polypeptide was synthesized on a hyper-acid-labile trityl resin that allows detachment from the resin under mild conditions with 30 % HFIP/CH_2_Cl_2_ to afford protected Pup(1–63) with a free C-terminal carboxylate available for coupling with an AMC-labeled glutamine analogue (Scheme 2). Therefore a suitably protected building block was synthesized starting from commercial Fmoc-Glu-O*t*Bu and AMC. The synthesis of coumarides is difficult because of the poor nucleophilicity of the aniline amino group and traditional peptide coupling methods using EDC/DMAP failed. However, when phosphoryl chloride was used for carboxyl activation, the Fmoc-Glu(AMC)-O*t*Bu product could be obtained, albeit in low yield of 21 %.^13^] Gratifyingly, formation of the acid chloride in situ under neutral conditions using Ghosez’s reagent,^14^] an α-chloroenamine, followed by the addition of AMC, led to a satisfying yield of 56 %. The Fmoc protecting group was removed with 50 % diethylamine in CH_2_Cl_2_ and the resulting H-Glu(AMC)-OH building block was reacted with protected Pup(1–63) in the presence of PyBOP and DIPEA as the condensing reagents. After deprotection and purification, the Pup-Glu(AMC) conjugate was obtained in high purity and 4 % overall yield (based on initial resin loading), the sequence was verified by trypsinolysis and tandem mass spectrometry (MS-MS) to give 100 % sequence coverage (see Supporting Information).

**Scheme 2:**
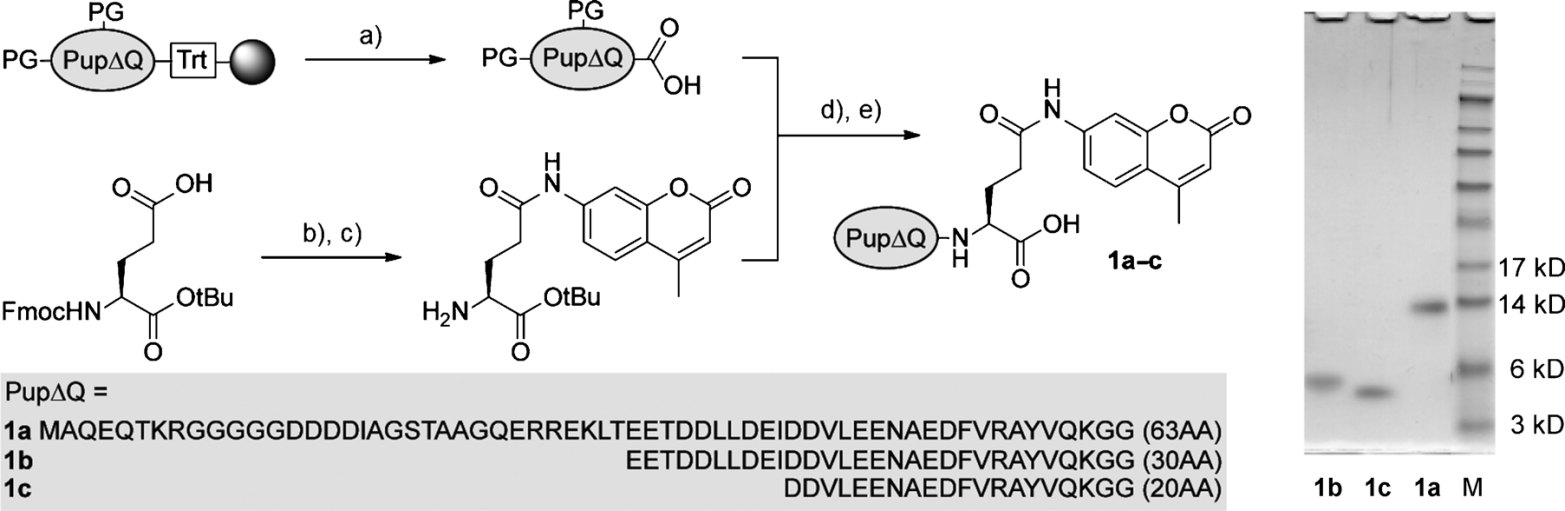
Chemical synthesis and SDS-PAGE (12 %) analysis of Pup-Glu(AMC) conjugates **1 a**–**c**. a) 30 % HFIP; b) Ghosez’s reagent, AMC; c) 50 % DEA; d) PyBOP, DIPEA; e) 95 % TFA. PG=protecting group; Trt=trityl; Fmoc=9-fluorenylmethyloxycarbonyl; HFIP=hexafluoroisopropanol; Ghosez’s reagent=1-chloro-*N*,*N*,2-trimethyl-1-propenylamine; AMC=7-amino-4-methylcoumarin; DEA=diethylamine; PyBOP=benzotriazol-1-yl-oxytripyrrolidinophosphonium hexafluorophosphate; DIPEA=*N*,*N*-diisopropylethylamine; TFA=trifluoroacetic acid.

The amino terminus of Pup is not essential for Dop activity.^15^] As smaller reagents are synthetically more accessible, we decided to synthesize two truncated Pup-Glu(AMC) analogues that contain 30 amino acids [Pup(33–63)-Glu(AMC), **1 b**] and 20 amino acids [Pup(43–63)-Glu(AMC), **1 c**] and have a Glu(AMC) residue attached to their C termini; these were compared to full-length Pup(1–63)-Glu(AMC) (**1 a**) in Dop assays (Scheme 2). All Pup-Glu(AMC) analogues were obtained in high purity and in similar overall yields (see Supporting Information). Each construct was tested as a substrate for Dop by monitoring the increase in fluorescence over time, representing cleavage of AMC from the C-terminal glutamine side chain of the Pup analogue. To confirm that the observed fluorescence originated from AMC cleavage, all reaction mixtures were also analyzed by mass spectrometry (see Supporting Information). Dop showed hydrolytic activity for both **1 a** and **1 b**, however, **1 c** was not a substrate for Dop (Figure 1 A). These results support the previous finding that the amino terminus of Pup is dispensable for Dop activity and that the Dop-binding region extends beyond the C terminus of Pup into the middle of the protein. DopE10A, a mutant proposed to be deficient in ATP binding, showed no activity with **1 a** (Figure 1 A). Also, Dop was unable to hydrolyze Ub-AMC, a substrate for deubiquitinases.

**Figure 1:**
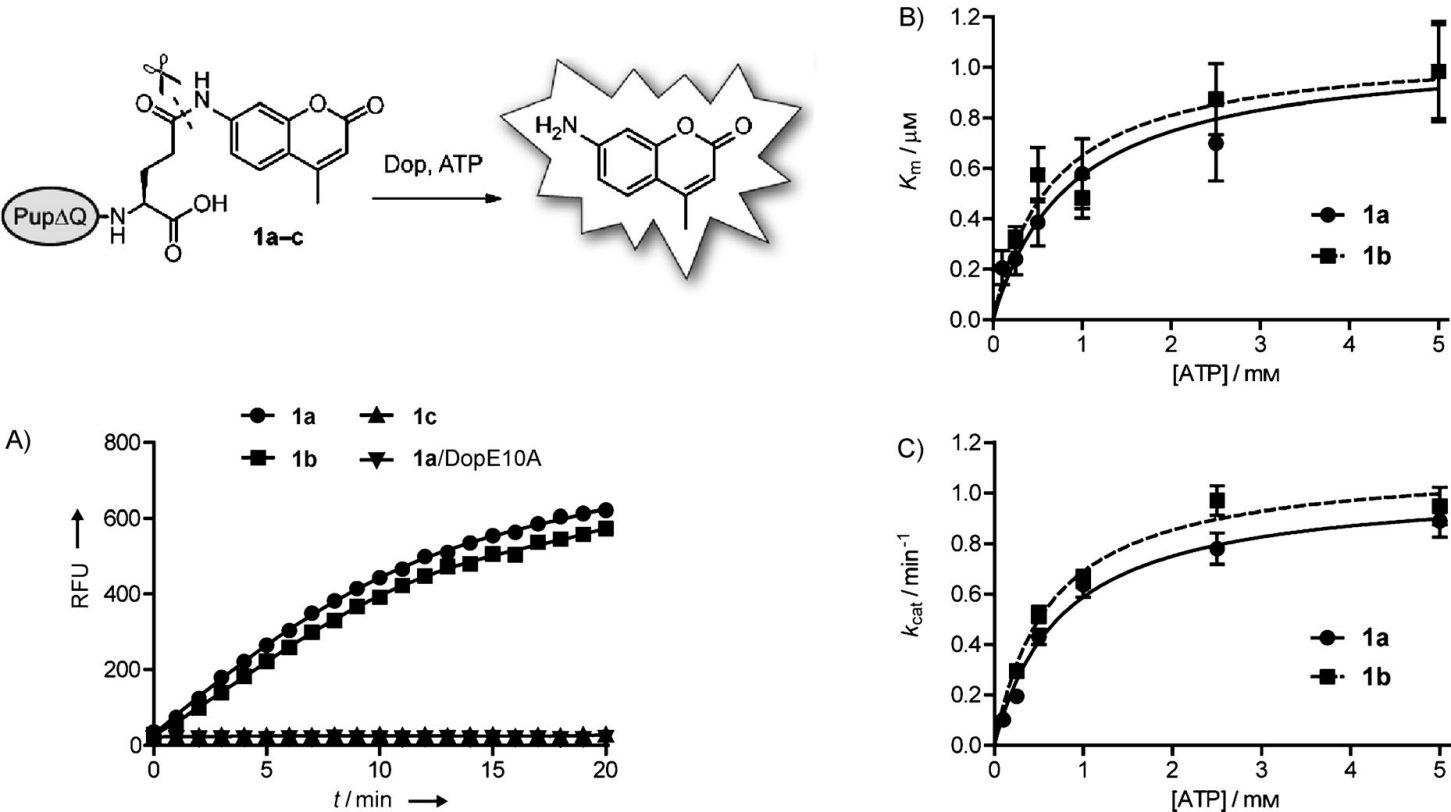
Dop activity assays. A) Analysis of Dop activity with Pup-Glu(AMC) conjugates 1 a–c. Assays were performed by monitoring the release of free AMC after treatment with Dop or the inactive DopE10A mutant. B) Measurement of *K*_m_ as a function of ATP concentration for Pup(1–63)-Glu(AMC) (1 a) and Pup(33–63)-Glu(AMC) (1 b) as the substrates. C) Measurement of *k*_cat_ as a function of ATP concentration for Pup(1–63)-Glu(AMC) (1 a) and Pup(33–63)-Glu(AMC) (1 b) as the substrates. RFU=relative fluorescence units.

The Dop substrates **1 a** and **1 b** were subjected to further analyses and their kinetic constants were determined at various concentrations of ATP (Figure 1 B and C). The data followed typical Michaelis–Menten kinetics and the *K*_m_ values were comparable with one another, to that of various deubiquitinating enzymes (UCH-L3=0.039 μm; IsoT=1.4 μm, determined using Ub-AMC as substrate), and to that of the Pup ligase PafA (*K*_m_=1.4 μm; determined using Pup_Glu_ as substrate).^4^^,^ ^11^] Despite relatively low *K*_m_ values, the *k*_cat_ values for Dop were also low (<1 min^−1^). A similar observation was also made for PafA, which has a *k*_cat_ value of 0.95 min^−1^ using Pup_Glu_ as substrate.^4^] Interestingly, Imkamp and colleagues found that Dop activity increased in the presence of the ATPase Mpa, which might interact with Pup, or Pup and Dop, to increase *k*_cat_ values in vivo.^6b^] In addition, it is also possible that the reaction kinetics were affected by the nature of our synthetic substrates, that is, AMC is a bulky hydrophobic group in comparison to the lysine side chain of a natural substrate.

Although Dop is an ATP-dependent enzyme, it does not require ATP hydrolysis for activity. Moreover, Dop can bind to Pup in the absence of ATP.^3^^,^ ^6a^] Our analysis revealed that ATP appears to function as an activator for the hydrolysis of substrates **1 a** and **1 b** by Dop, as increasing the ATP concentration resulted in an increase in catalytic efficiency for both substrates (Table 1). The maximum catalytic efficiency was reached at ATP concentrations between 0.5–2.5 mm (Table 1).

**1:** Catalytic efficiency *k*_cat_/*K*_m_ [×10^3^ m^−1^ s^−1^] at different concentrations of ATP.

[ATP] / mm	**1 a**	**1 b**
0.10	8.29±2.8	not determined
0.25	13.5±3.6	15.0±1.9
0.50	18.6±4.6	15.0±2.9
1.00	18.3±4.6	22.8±3.9
2.50	18.6±4.2	18.5±3.2
5.00	15.1±3.0	16.1±3.5

To investigate the specificity of our synthetic Dop substrates, we monitored the hydrolysis of **1 a** in lysates of Mtb as well as lysates of *Escherichia coli* (Figure 2). Lysates from the wild type (WT)*, pafA*-null mutant, and complemented *dop*-null mutant Mtb strains showed hydrolysis of **1 a**. In contrast, lysates from *dop* mutant, the *dop* mutant complemented with the *dopE10A* allele, or *E. coli* could not hydrolyze **1 a**. These results illustrate the high specificity of **1 a** for Dop, even in lysates.

**Figure 2:**
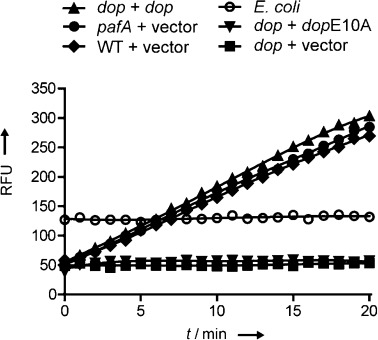
Hydrolytic activity in lysates of various Mtb and *E. coli* strains using Pup(1–63)-Glu(AMC) (1 a) as the substrate. Lysates of *pafA* (green) and *dop* (grey, red, blue) mutants containing empty plasmid vector or vector with indicated genes were analyzed along with lysates from WT Mtb (purple) with empty vector and lysates of *E. coli* (orange) for hydrolysis of Pup(1–63)-Glu(AMC) (1 a). RFU=relative fluorescence units.

In summary, we have developed new Pup-based fluorogenic substrates with high specificity towards hydrolysis by Dop. Moreover, **1 b**, an amino-terminus-truncated analogue of Pup, performed equally well as the full length Pup-Glu(AMC) conjugate (**1 a**), however, Dop did not show any activity toward the shorter Pup(43–63)-Glu(AMC) (**1 c**) conjugate. As Dop might be considered as an attractive new drug target for Mtb, the fluorogenic substrates developed here might find application as high-throughput screening assay reagents for the identification of Dop inhibitors.

## Experimental Section

**Synthesis of peptides 1a–c:** The PupΔQ sequence was synthesized on a preloaded Fmoc-amino-acid trityl resin (0.2 mmol g^−1^) at 25 μmol scale, using fourfold excess of appropriately side-chain-protected Fmoc-amino acids in NMP relative to the resin; PyBOP (4 equiv) and DIPEA (8 equiv) were used as condensing reagents. Fmoc removal was carried out using 20 % piperidine in *N*-methyl-2-pyrrolidone (NMP) for 2×2 min and 1×5 min and capping of the resin was performed with a mixture of Ac_2_O/DIPEA/HOBt (500, 125 and 15 mm respectively in NMP, 3×1.2 mL, 2×2 min and 1×5 min). During the synthesis different coupling protocols were used: coupling cycle 1–30: single couplings of 40 min, double couplings of 2×40 min for cycles 21, 22, 24 and 28, no capping; coupling cycle 31–39: single couplings of 60 min, double couplings of 2×60 min for cycles 33, 34, 38 and 39, no capping; coupling cycle 40–61: single couplings of 60 min, double couplings of 2×60 min for cycles 40, 42, 46, 48, 51–54, 58 and 59, capping after each coupling cycle. During the chain elongation steps, dipeptide pseudoproline building blocks were incorporated at positions 21/22 (Fmoc-Ser-Thr(Ψ^Me,Me^pro)-OH) and 32/33 (Fmoc-Leu-Thr(Ψ^Me,Me^pro)-OH. After completion of the SPPS, the resin was washed with Et_2_O, dried under high vacuum and stored for further use.

Then, the resin bound polypeptide was treated with 5 mL of CH_2_Cl_2_/HFIP (7:3, *v*/*v*) for 30 min and filtered. This CH_2_Cl_2_/HFIP treatment was repeated once more and the resin was rinsed with CH_2_Cl_2_ (3×5 mL). The combined filtrates were concentrated, co-evaporated with CH_2_Cl_2_ and dried under high vacuum. The partially protected peptide residue (1 equiv) was dissolved in CH_2_Cl_2_ and treated with H-Glu(AMC)-O*t*Bu (45 mg, 125 μmol, 5 equiv) in the presence of PyBOP (65 mg, 125 μmol, 5 equiv) and TEA (35 μL, 250 μmol, 10 equiv). The reaction mixture was stirred overnight at room temperature. The volatiles were removed in vacuo and the residue was treated with TFA/H_2_O/triisopropylsilane (TIS; 95:2.5:2.5, *v*/*v*/*v*) for 3 h followed by precipitation with ice-cold Et_2_O/pentane (3:1, *v*/*v*). The precipitated crude protein was washed with Et_2_O/pentane (3:1, *v*/*v*, once) and Et_2_O (twice). Finally, the pellet was dissolved in a mixture of H_2_O/CH_3_CN/HOAc (65:25:10, *v*/*v*/*v*) and lyophilized. The crude product was purified by preparative HPLC on a Waters Atlantis prep T3 column (10×150 mm, 5 μm), using two mobile phases: solvent A=0.1 % aq. TFA and solvent B=0.1 % formic acid in CH_3_CN with the following gradient: 0–5 min: 5 % B; 5–8 min→25 % B; 8–30 min→60 % B; 30–33 min→95 % B; 33–35 min: 95 % B.

**MAQEQT KRGGGG GDDDDI AGSTAA GQERRE KLTEET DDLLDE IDDVLE ENAEDF VRAYVQ KGG-Glu(AMC) (1 a):** Yield: 4.3 mg (4 %), *R*_t_:7.08 min (analytical HPLC-MS was performed on a Waters Alltima C18 column (2.1×100 mm, 3 μm) using two mobile phases: solvent A=0.1 % aq. formic acid and solvent B=0.1 % formic acid in CH_3_CN under the following conditions: flow rate=0.4 mL min^−1^, runtime=20 min, column *T*=40 °C. Gradient: 0–1 min: 5 % B; 1–11 min: → 95 % B; 11–16 min: 95 % B), MS (ESI^+^) *m*/*z* calcd: 7098 [*M*]; found: 7099 [*M*+H]^+^.

**EETDDL LDEIDD VLEENA EDFVRA YVQKGG-Glu(AMC) (1 b):** Yield: 2.1 mg (5 %), *R*_t_:7.78 min (analytical HPLC-MS was performed in the same way as for **1 a**), MS (ESI+) *m*/*z* calcd: 3711 [*M*]; found: 3711 [*M*+H]^+^.

**DDVLEE NAEDFV RAYVQK GG-Glu(AMC) (1 c):** Yield: 4.3 mg (4 %), *R*_t_:6.62 min (analytical HPLC-MS was performed in the same way as for **1 a**), MS (ESI+) *m*/*z* calcd: 2538 [*M*]; found 2539 [*M*+H]^+^.

**H-Glu(AMC)-O*t*Bu:** Synthetic details on the preparation of H-Glu(AMC)-O*t*Bu and characterization data are included in the Supporting Information.

**Photometric assays:** Reactions contained Dop-His_6_ (3–3.5 μg),6a substrate (2 μm), ATP (2.5 mm unless otherwise indicated), MgCl_2_ (20 mm), DTT (1 mm), and NaCl (50 mm) in Tris (50 mm, pH 8) in a final volume of 100 μL in a 96-well plate format. Reactions were monitored by measuring the increase in fluorescence emission at 460 nm (*λ*_ex_=355 nm) that correlated with hydrolysis of AMC from the substrate on a SpectraMax M5 (Molecular Devices) spectrophotometer. SoftMax Pro (Molecular Devices) was used to record the data and GraphPad Prism was used to fit the kinetic data.

For Mtb lysate experiments, Mtb were grown to OD_580_=1–1.2, at which time 50 OD equivalents were harvested, washed with 0.05 % Tween-80 in PBS (25 mL). The cells were resuspended in Tris [100 mm, EDTA (1 mm), pH 8, 1 mL] and transferred to bead-beating tubes with zirconia silica beads (250 μL). Cells were lysed by bead-beating three times for 30 s each time. Lysates were filtered through 0.45 μm filters, glycerol was added (12 % final volume) and the samples were either analyzed immediately or stored at −20 °C for further use. Lysate reactions (final volume of 100 μL) contained lysate (40 μL), substrate **1 a** (3 μm), ATP (5 mm), MgCl_2_ (20 mm), DTT (1 mm), 1× energy regeneration solution (Boston Biochem) and NaCl (50 mm) in Tris (50 mm, pH 8).

**Mass spectrometric assays:** To a solution of peptide **1 a**, **1 b**, **1 c** or Ub-AMC (3.3 μm) in assay buffer [Tris (50 mm, pH 8) containing ATP (2.5 mm), MgCl_2_ (20 mm), DTT (1 mm), and NaCl (50 mm)], Dop-His_6_6a was added and the mixture was incubated at RT for 2 h. As a control, a solution of the peptide (3.3 μm) in assay buffer without added Dop-His_6_ was incubated at RT for 2 h. After 2 h, CH_3_CN (60 μL) was added to both reaction vials, the samples were centrifuged at 16 000 *g* for 5 min. Aliquots (25 μL) were taken from each mixture for ESI MS analysis.

## Notes

Detailed facts of importance to specialist readers are published as “Supporting Information”. Such documents are peer-reviewed, but not copy-edited or typeset. They are made available as submitted by the authors.
