# Supplementary material for: Synthesis and Evaluation of a Selective Fluorogenic Pup Derived Assay Reagent for Dop, a Potential Drug Target in Mycobacterium tuberculosis
Source: Chembiochem. 2012 Aug 24;13(14):2056–60. doi: 10.1002/cbic.201200460 (PMC3474595; doi:10.1002/cbic.201200460)
Supplement: Supplementary file 1 [file cbic0013-2056-sd1.pdf]

CHEMBIOCHEM

Supporting Information

© Copyright Wiley-VCH Verlag GmbH & Co. KGaA, 69451 Weinheim, 2012

Synthesis and Evaluation of a Selective Fluorogenic Pup Derived Assay Reagent for Dop, a Potential Drug Target in *Mycobacterium tuberculosis*

Remco Merkx,[a] Kristin E. Burns,[b] Paul Slobbe,[a] Farid El Oualid,[a] Dris El Atmioui,[a] K. Heran Darwin,[b] and Huib Ovaa*[a]

cbic_201200460_sm_miscellaneous_information.pdf

SUPPORTING INFORMATION

1 General info

General reagents were obtained from Sigma Aldrich, Fluka and Acros and used as received. Fmoc-Glu-OtBu was purchased from Chemimpex International, Ghosez' reagent (1-Chloro-N,N,2-trimethyl-1-propenylamine) was obtained from Sigma Aldrich. Solvents were purchased from BIOSOLVE and, where necessary, dried over molecular sieves (4Å for DCM). Dry pyridine and DMF were obtained from Acros. Peptide synthesis reagents were purchased from Novabiochem. Photometric Dop assays were monitored on a SpectraMax M5 (Molecular Devices) spectrophotometer operated through SoftMax Pro (Molecular Devices) software by measuring the increase in fluorescence emission at 460nm (λex = 355nm), GraphPad Prism was used to fit the kinetic data. Analytical thin layer chromatography was performed on aluminium sheets precoated with silica gel 60 F_{254}, spots were visualized using 20% ninhydrin in ethanol and heating by a heatgun. Column chromatography was carried out on silica gel (0.035-0.070 mm, 90Å, Acros). Nuclear magnetic resonance spectra (^{1}H-NMR, ^{13}C-NMR and COSY) were determined in DMSO-d_6 (^{1}H δ 2.50 ppm; ^{13}C δ 39.5 ppm) using a Bruker ARX 400 Spectrometer (^{1}H: 300 MHz, ^{13}C: 100 MHz) at 298 K. Peak shapes in NMR spectra are indicated with the symbols 'd' (doublet), 'dd' (double doublet), 's' (singlet), 't' (triplet) and 'm' (multiplet). Chemical shifts (δ) are given in ppm and coupling constants *J* in Hz. LC-MS measurements were performed on a system equipped with a Waters 2795 Seperation Module (Alliance HT), Waters 2996 Photodiode Array Detector (190-750nm), Waters Alltima C18 (2.1x100mm, 3 µm) column, Phenomenex Kinetex C18 (2.1x50, 2.6 µm) column and LCT™ Orthogonal Acceleration Time of Flight Mass Spectrometer. Samples were run using 2 mobile phases: A = 1% CH_3CN, 0.1% formic acid in water and B = 1% water and 0.1% formic acid in CH_3CN. Data processing was performed using Waters MassLynx Mass Spectrometry Software 4.1 (deconvulation with Maxent1 function). Pereparative HPLC was performed on a Shimadzu Prominence HPLC system equipped with a Waters Atlantis prep T3™ (10x150mm, 5 µm) column, using 2 mobile phases: A = 0.1% TFA in water and B = 0.1% formic acid in CH_3CN. Nano-LC-MSMS was performed on a Dionex U3000 nano system coupled to a Thermo LTQ Orbitrap XL Mass Spectrometer using a Top 5 instrument method (1 x FT-MS scan (mz range 335-1800 ; 60K resolution) and 5 x IT MSMS scans) over 40 min (5 → 40% B), using 2 mobile phases: A = 0.1% formic acid in water and B = 10% water, 0.1% formic acid in CH_3CN. Results were analyzed with Thermo Scientific Proteome Discoverer 1.3 software.

LC-MS methods

Method 1: Waters Alltima C18, 2.1×100 mm, 3 µm); flow rate = 0.4 mL/min, runtime = 20 min, column T = 40°C. Gradient: 0 − 1 min: 5% B; 1 − 11 min: → 95% B; 11 − 16 min: 95% B.

Method 2: Phenomenex Kinetex C18, (2.1×50 mm), 2.6 µm); flow rate = 0.8 mL/min, runtime = 6 min, column T = 40°C. Gradient: 0 − 0.5 min: 5% B; 0.5 − 4 min: → 95% B; 4 −5.5 min: 95% B.

Preperative HPLC method

Flow rate = 7.5 mL/min, runtime 35 min, column T = 40°C. Gradient: 0 – 5 min: 5% B; 5 – 8 min: → 25% B; 8 –30 min: → 60% B; . 30 –33 min: → 95% B; 33 –35 min: 95% B.

General Fmoc SPPS Strategy

SPPS was performed on a Syro II MultiSyntech Automated Peptide synthesizer using standard 9-fluorenylmethoxycarbonyl (Fmoc) based solid phase peptide chemistry at 25 µmol scale, using fourfold excess of amino acids in NMP relative to pre-loaded Fmoc amino acid trityl resin (0.2 mmol/g, Rapp Polymere GmbH), PyBOP (4 equiv) and DIPEA (8 equiv) were used as condensing reagents. All amino acids were Fmoc protected, except for the final N-terminal amino acids which were introduced as the corresponding Boc derivative. The following protected amino acid and pseudoproline dipeptide building blocks were used during Pup synthesis: Fmoc-L-Ala-OH, Fmoc-L-Arg-(Pbf)-OH, Fmoc-L-Asn(Trt)-OH, Fmoc-L-Asp(O*t*Bu)-OH, Fmoc-L-Gln(Trt)-OH, Fmoc-L-Glu(OtBu)-OH, Fmoc-L-Gly-OH, Fmoc-L-Ile-OH, Fmoc-L-Leu-OH, Fmoc-L-Lys(Boc)-OH, Fmoc-L-Phe-OH, Fmoc-L-Ser(*t*Bu)-OH, Fmoc-L-Thr(*t*Bu)-OH, Fmoc-L-Tyr(*t*Bu)-OH, Fmoc-L-Val-OH, Boc-L-Asp(OtBu)-OH, Boc-L-Glu(OtBu)-OH, Boc-L-Met-OH, Fmoc-Ser-Thr($\Psi^{Me,Me}$pro)-OH) and Fmoc-Leu-Thr($\Psi^{Me,Me}$pro)-OH. All amino acid and dipeptide building blocks were dried under high vacuum overnight prior to use. Fmoc removal was carried out using 20% piperidine in NMP for 2 × 2 and 1 × 5 min. Capping of the resin was performed with a mixture of Ac_2O/DIPEA/HOBt in NMP at 500 mM, 125 mM and 15 mM respectively (3 × 1.2 mL, 2 × 2 and 1 × 5 min). This solution was prepared freshly on ice every 2 days.

Coupling cycle 1 – 30:

- Single couplings of 40 min
- Double couplings of 2 × 40 min only for cycles 21, 22, 24 and 28
- No capping

Coupling cycle 31 – 39:

- Single couplings of 60 min
- Double couplings of 2 × 60 min only for cycles 33, 34, 38 and 39
- No capping

Coupling cycle 40 – 61

- Single couplings of 60 min
- Double couplings of 2 × 60 min only for cycles 40, 42, 46, 48, 51 – 54, 58 and 59
- Capping after each coupling cycle

After completion of the synthesis, the resin was washed with Et_2O, dried under high vacuum and stored for further use.

Use of dipeptide pseudoproline building blocks

Three Pup(1-63) sequences were synthesized using one (B), two (C) or no (A) pseudoproline dipeptide building block(s), the crude products were analyzed by SDS page and HPLC-MS (method 2). The position of the pseudoprolines in the Pup sequence is marked with an underscore. Introduction of 2 pseudoproline dipeptide building blocks largely prevented formation of side products which arise from terminations and deletions.

A	MAQEQTKRGGGGGDDDDIAGSTAAGQERREKLTEETDDLLDEIDDVLEENAEDFVRAYVQKGG
B	MAQEQTKRGGGGGDDDDIAGSTAAGQERREKLTEETDDLLDEIDDVLEENAEDFVRAYVQKGG
C	MAQEQTKRGGGGGDDDDIAGSTAAGQERREKLTEETDDLLDEIDDVLEENAEDFVRAYVQKGG

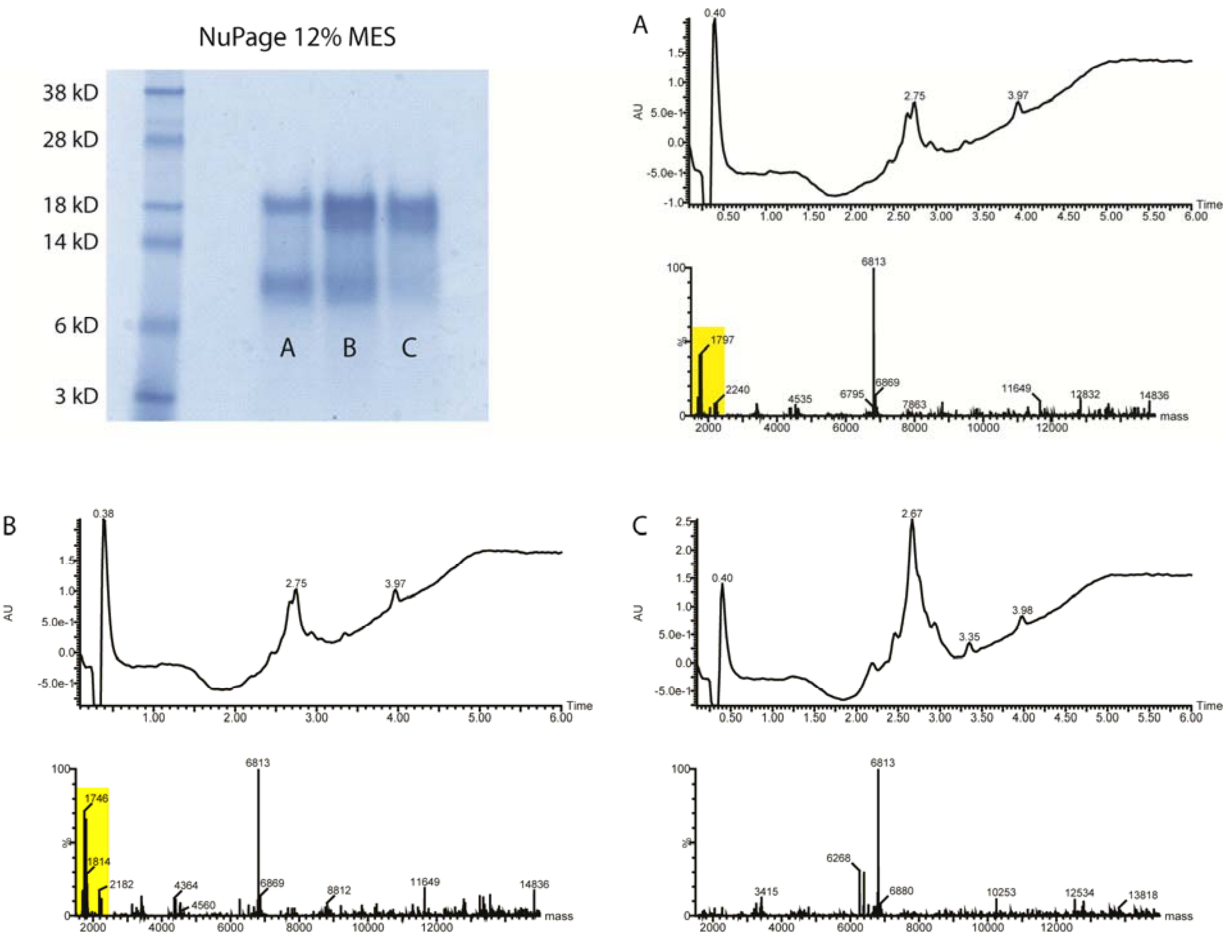

2. Synthesis of H-Glu-OtBu

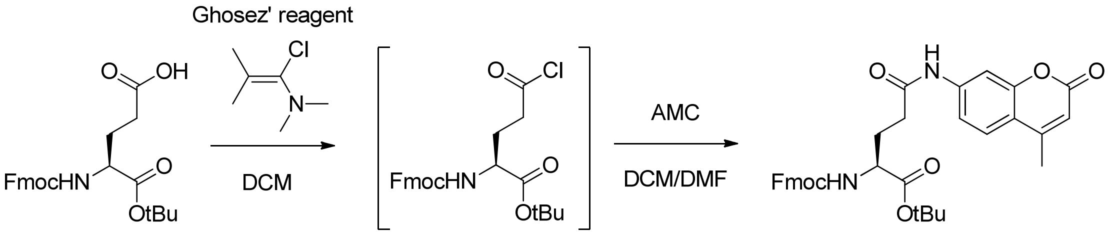

Fmoc-Glu-OtBu **2**

Fmoc-Glu(AMC)-OtBu (2). Under argon, 1-Chloro-N,N,2-trimethyl-1-propenylamine (Ghosez' reagent) (440 µL, 3.3 mmol) was added to a cooled (0°C) and stirred solution of Fmoc-Glu(AMC)-OtBu (700.0 mg, 1.6 mmol) in dry DCM (10 mL). The resulting mixture was immediately transferred to a separate flask containing a stirred solution of AMC (316.0 mg, 1.8 mmol) in dry DMF (40 mL) and the resulting mixture was stirred at room temperature under argon. After 16 h an extra portion of the Ghosez' reagent (220 µL, 1.65 mmol) was added and stirring was continued for one additional hour to ensure complete conversion of the Fmoc-Glu-OtBu starting material, as was confirmed by HPLC-MS analysis (program 2). Volatiles were removed *in vacuo* and the residue was taken up in EtOAc (50 mL) and washed with 1N $KHSO_4$ (3 × 50 mL) and Brine (3 × 50 mL) and dried (Na_2SO_4). The product was isolated by flash column chromatography (eluent: Et_2O) as a white foam (560 mg, 58%). R_f: 0.2 (eluent: Et_2O), R_t: 3.70 min (method 2), MS ES+ (amu) calculated: 582.24 [M]; found: 583.34 $[M+H]^+$, 605.33 $[M+Na]^+$, 527.22 $[(M–tBu)+H]^+$; ^1H NMR (300 MHz, $CDCl_3$) δ 9.19 (1H, s), 8.51 (1H, d, *J* = 8.1 Hz), 7.73 (3H, m), 7.59 (2H, m), 7.49 (1H, *J* = 8.7 Hz), 7.37 (2H, m), 7.27 (2H, m), 6.17 (1H, s), 5.87 (1H, d, *J* = 8.1 Hz), 4.47 (1H, m), 4.40 (2H, m), 4.18 (1H, t, *J* = 6.2 Hz), 2.52 (2H, m), 2.38 (3H, m), 2.04 (1H, m), 1.49 (9H, br s); ^{13}C NMR (100 MHz, $CDCl_3$) δ 171.3, 171.0, 161.7, 156.8, 154.0, 153.0, 143.7 (double line), 142.3, 141.3, 127.7 (double line), 127.1 (double line), 125.3, 125.1, 125.0, 120.0 (double line), 115.8 (double line), 112.9, 106.7, 82.8, 67.1, 54.0, 47.2, 34.0, 29.4, 28.0, 18.5.

Alternative procedure for the synthesis of ***2*** *using* $POCl_3$*:*

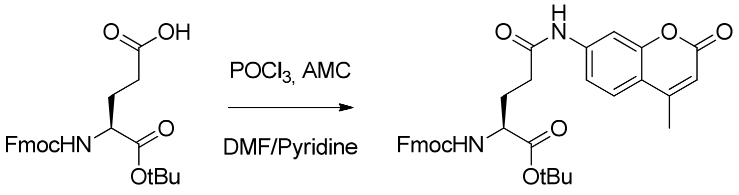

Fmoc-Glu-OtBu **2**

Fmoc-Glu(AMC)-OtBu (2). Under Nitrogen, $POCl_3$ (504 µL, 5.4 mmol) was added dropwise to a cooled (-15°C) and stirred mixture of AMC (0.87g, 4.9 mmol) and Fmoc-Glu(AMC)-OtBu (2.0 g, 4.7 mmol) in dry DMF/Pyridine (2:1 v/v, 100 mL). The reaction mixture was allowed to warm up to room temperature and

stirring was continued for 16 h before the solvents were removed *in vacuo*. The residu was coevapporated with toluene and taken up in EtOAc (300 mL), washed with 1N $KHSO_4$ (2 × 200 mL) and Brine (2 × 200 mL) and dried (Na_2SO_4). The product was isolated by flash column chromatography (eluent: Et_2O) as a white foam (600 mg, 21%).

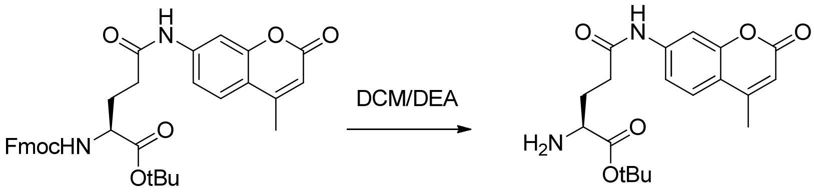

2 **3**

H-Glu(AMC)-OtBu (3). A portion of **2** (200 mg 0.34 mmol) was dissolved in diethylamine/dicholoromethane (1:1 v/ v, 16 mL) and stirred at room temperature. After 3h the reaction mixture was concentrated *in vacuo*. The product was isolated by flash column chromatography (eluent: DCM → 5% MeOH in DCM) as a white solid (92 mg, 75%). R_f: 0.2 (5% MeOH in DCM); R_t = 2.22 min (method 2), MS ES+ (amu) calculated: 360.17 [M]; found 360.05 $[M+H]^+$, 305.05 $[(M\text{–}tBu)+H]^+$; ^{1}H-NMR (300 MHz, DMSO-d_6) δ 10.38 (s, 1H), 7.76 (1H, d, J = 2.0 Hz), 7.70 (1H d, J = 8.7 Hz), 7.47 (1H dd, J = 2.0, 8.7 Hz), 6.25 (1H, d, J = 1.2 Hz), 3.23 (1H, m), 2.48 (2H, m, *partially under DMSO peak*), 2.40 (3H, s), 1.91 (1H, m), 1.80 (2H, m), 1.69 (1H, m), 1.40 (9H, s); ^{13}C NMR (100 MHz, DMSO-d_6) 174.9, 171.7, 160.0, 153.7, 153.1, 142.6, 125.8, 115.0, 114.7, 112.1, 105.4, 79.9, 54.0, 32.8, 29.7, 27.7, 17.9.

3. Synthesis of and characterization compounds 1a – c and Ub-AMC

General method for the C-terminal modification of PupΔQ (1)

The PupΔQ sequence was synthesized on a trityl resin following the general procedure. Then, the resin bound polypeptide was treated with 5 mL of DCM/HFIP (7:3 v/v) for 30 min and filtered. This DCM/HFIP treatment was repeated once more and the resin was rinsed with DCM (3 × 5 mL). The combined filtrates were concentrated, coevapporated with DCM and dried under high vacuum. The partially protected peptide residue (1 equiv) was redissolved in DCM and reacted with H-Glu(AMC)-O*t*Bu (45 mg, 125 µmol, 5 equiv) in the presence of PyBOP (65 mg, 125 µmol, 5 equiv) and TEA (35 µL, 250 µmol, 10 equiv). The reaction mixture was stirred over night at room temperature. The volatiles were removed *in vacuo* and the residue was treated with TFA/H_2O/TiS (95:2.5:2.5 v/v/v) for 3 h followed by precipitation with cold Et_2O/pentane (3:1 v/v). The precipitated crude protein was washed with Et_2O/pentane (3:1 v/v, once) and Et_2O (twice). Finally, the pellet was dissolved in a mixture of H_2O/CH_3CN/HOAc (65/25/10 v/v/v) and lyophilized. The crude product was purified by preparative HPLC.

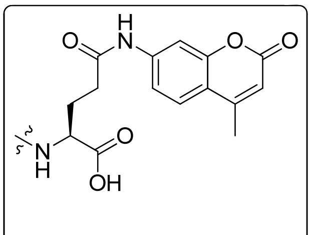

MAQEQTKRGGGGGDDDDIAGSTAAGQERREKLTEETDDLLDEIDDVLEENAEDFVRAYVQKGG-Glu(AMC) (1a). Yield: 4.3 mg (4%), R_t:7.08 min (method 1), MS ES+ (amu) calculated: 7098 [M]; found 7099 $[M+H]^+$. The chemical integrity of this compound was further confirmed by MSMS analysis, 100% coverage was found including the AMC-modified C terminus of the peptide.

EETDDLLDEIDDVLEENAEDFVRAYVQKGG-Glu(AMC) (1b). Yield: 2.1 mg (5%), R_t:7.78 min (method 1), MS ES+ (amu) calculated: 3711 [M]; found 3711 $[M+H]^+$.

DDVLEENAEDFVRAYVQKGG-Glu(AMC) (1c). Yield: 4.3 mg (4%), R_t:6.62 min (method 1), MS ES+ (amu) calculated: 2538 [M]; found 2539 $[M+H]^+$.

Ub-AMC. The synthesis of ubiquitin-AMC was performed following the procedure as was described earlier.[1]

4. Dop activity assays

Photometric assays

Reactions contained Dop-His$_6$ (3-3.5 μg),[2] substrate (2 μM), ATP (2.5 mM unless otherwise indicated), $MgCl_2$ (20 mM), DTT (1 mM), and NaCl (50 mM) in Tris (50 mM, pH8) in a final volume of 100 μL in a 96-well plate format. Reactions were monitored by measuring the increase in fluorescence emission at 460nm (λex = 355 nm) that correlates with hydrolysis of AMC from the substrate.

For *Mtb* lysate experiments, *Mtb* were grown to an OD_{580}= 1 – 1.2 after which 50 OD equivalents were harvested and washed with 25 mL of 0.05% Tween-80 in PBS. The cells were resuspended in 1 mL of 100 mM Tris, pH8, 1 mM EDTA and transferred to bead beating tubes with 250 μL of zirconia silica beads. Cells were lysed by bead beating three times for 30 sec each time. Lysates were filtered through 0.45 μm filters, glycerol was added to 12% final volume. Lysate samples were either used immediately or stored at -20°C for further use. Lysate reactions contained 40 μL lysate, 3 μM substrate **1a**, 5 mM ATP, 20 mM $MgCl_2$, 1 mM DTT, 1 x energy regeneration solution (Boston Biochem) and 50 mM NaCl in 50 mM Tris, pH8 in a final volume of 100 μL.

Michaelis Menten analysis using Pup(1-63)-Glu(AMC) (1a) as the substrate

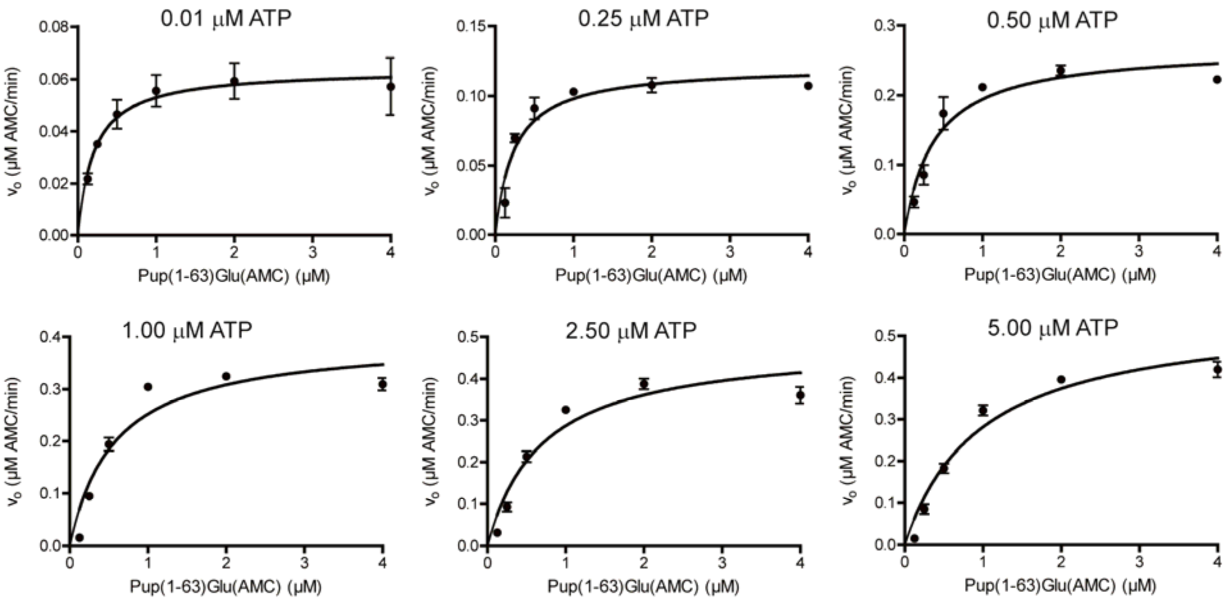

ATP (mM)	k_{cat} (min^{-1})	Std. Error	K_m (μM)	Std. Error	k_{cat}/K_m (MS^{-1})	Std. Error
0,10	1,02E-01	7,40E-03	2,05E-01	6,78E-02	8,29E+03	2,81E+03
0,25	1,94E-01	1,20E-02	2,40E-01	6,20E-02	1,35E+04	3,58E+03
0,50	4,30E-01	3,00E-02	3,86E-01	9,30E-02	1,86E+04	4,66E+03
1,00	6,37E-01	5,00E-02	5,79E-01	1,39E-01	1,83E+04	4,63E+03
2,50	7,80E-01	6,29E-02	7,00E-01	1,50E-01	1,86E+04	4,24E+03
5,00	8,90E-01	6,46E-02	9,83E-01	1,86E-01	1,51E+04	3,05E+03

Michaelis-Menten analysis using Pup(33-63)-Glu(AMC) (1b) as the substrate

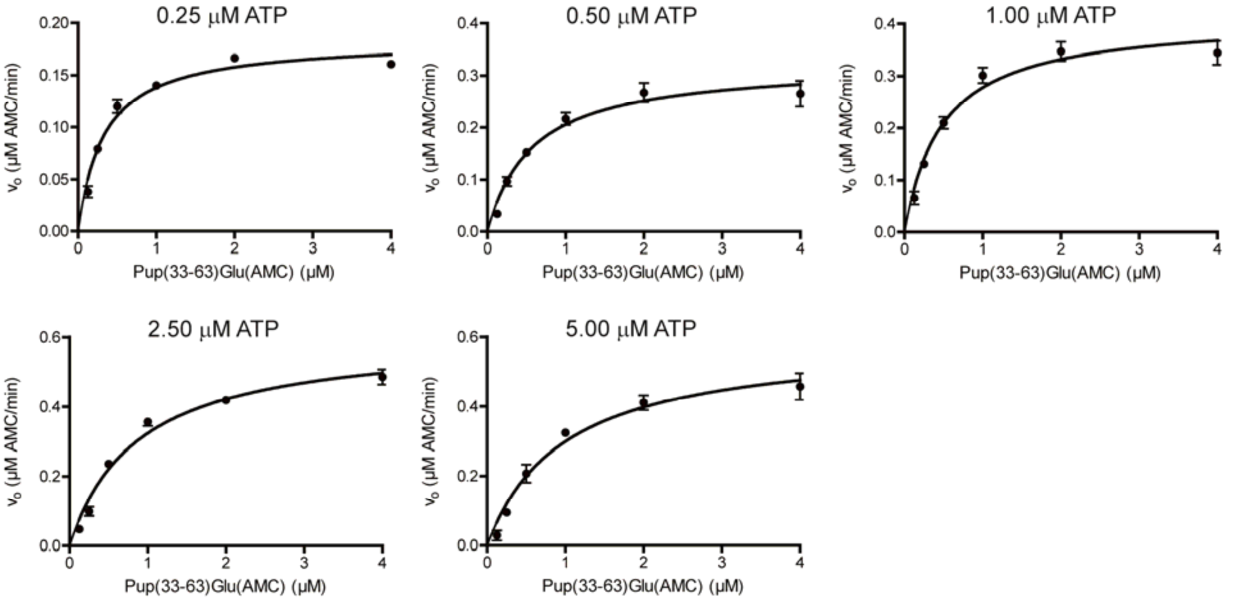

ATP (mM)	k_{cat} (min^{-1})	Std. Error	K_m (μM)	Std. Error	k_{cat}/K_m (MS^{-1})	Std. Error
0,25	2,94E-01	1,05E-02	3,27E-01	4,10E-02	1,50E+04	1,95E+03
0,50	5,18E-01	3,17E-02	5,74E-01	1,08E-01	1,50E+04	2,97E+03
1,00	6,60E-01	3,39E-02	4,82E-01	8,01E-02	2,28E+04	3,97E+03
2,50	9,70E-01	5,77E-02	8,74E-01	1,41E-01	1,85E+04	3,18E+03
5,00	9,48E-01	7,32E-02	9,83E-01	1,98E-01	1,61E+04	3,47E+03

Mass spectrometric assays

To a solution of peptide **1a**, **1b**, **1c** or Ub-AMC (3.3 µM) in assay buffer (containing: 2.5 mM ATP, 20 mM $MgCl_2$, 1 mM DTT, and 50 mM NaCl in 50 mM Tris, pH8), Dop-His_6 was added and the mixture was incubated at RT for 2 h. As a control, a solution of the peptide (3.3 µM) in assay buffer without added Dop-His_6 was incubated at RT for 2 h. After 2 h 60 µL CH_3CN was added to both reaction vials, the samples were centrifuged at 13.000 rpm for 5 min. Aliquots (25 µL) were taken from each mixture for ESMS analysis.

Table S1 Sequence and calculated molecular mass for the different peptides used in the Dop assay

**Peptide**	**Sequence**	**MS ES+ (amu) calculated**
**1a**	MAQEQTKRGG GGGDDDDIAG STAAGQERRE KLTEETDDLL DEIDDVLEEN AEDFVRAYVQ KGG-Glu(AMC)	7098
**1a'**	MAQEQTKRGG GGGDDDDIAG STAAGQERRE KLTEETDDLL DEIDDVLEEN AEDFVRAYVQ KGGE	6941
**1b**	EETDDLLDEI DDVLEENAED FVRAYVQKGG-Glu(AMC)	3712
**1b'**	EETDDLLDEI DDVLEENAED FVRAYVQKGGE	3555
**1c**	DDVLEENAED FVRAYVQKGG-Glu(AMC)	2538
Ub-AMC	MQIFVKTLTG KTITLEVEPS DTIENVKAKI QDKEGIPPDQ QRLIFAGKQL EDGRTLSDYN IQKESTLHLV LRLRGG-AMC	8717

Table S2 Found molecular masses after incubation in the Dop assay

**Peptide**	**Incubated with Dop MS ES+ (amu) found**	**Incubated without Dop MS ES+ (amu) found**	**Hydrolysis**
Pup(1-63)-Glu(AMC) (**1a**)	6941	7098	Yes
Pup(33-63)-Glu(AMC) (**1b**)	3555	3713	Yes
Pup(43-63)-Glu(AMC) (**1c**)	2539	2539	No
Ub-AMC	8717	8717	No

Pup(1-63)-Glu(AMC) (1a) with DOP

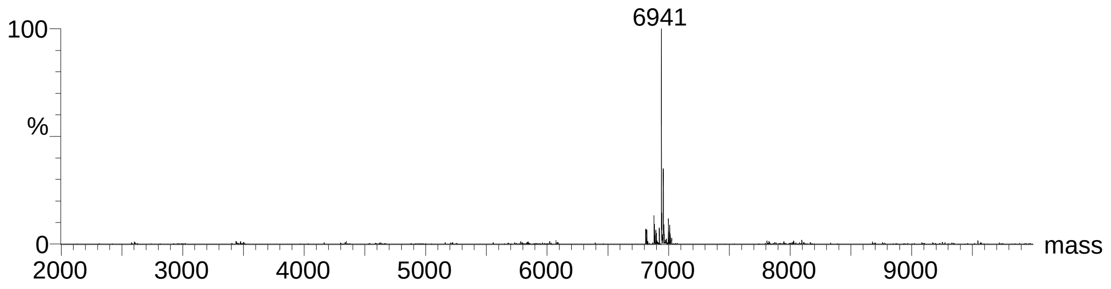

Pup(1-63)-Glu(AMC) (1a) without DOP

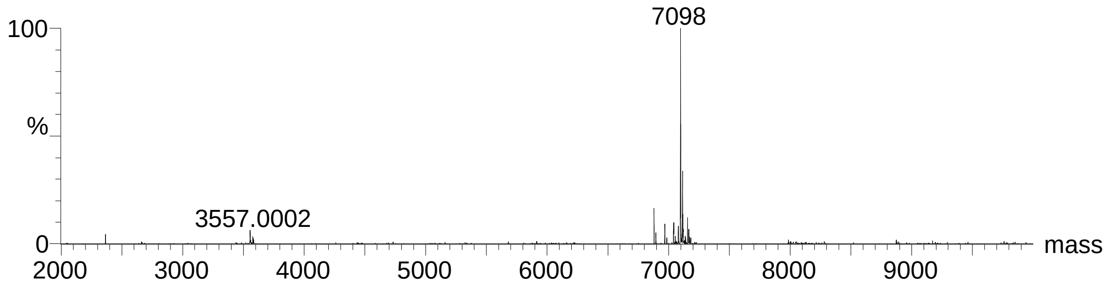

Pup(33-63)-Glu(AMC) (1b) without DOP

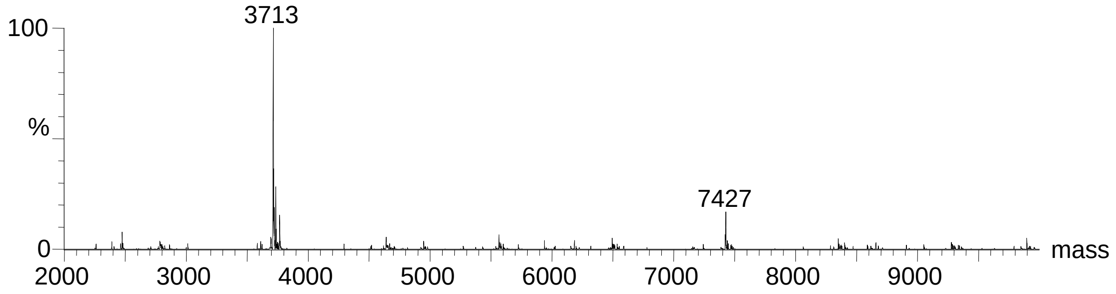

Pup(33-63)-Glu(AMC) (1b) with DOP

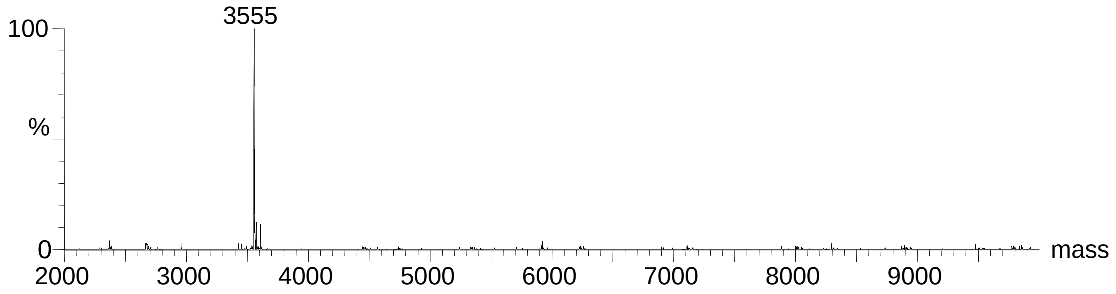

Pup(43-63)-Glu(AMC) (1c) with DOP

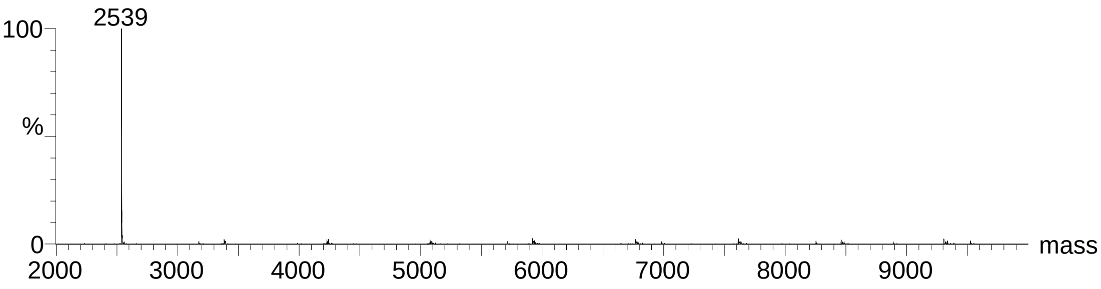

Pup(43-63)-Glu(AMC) (1c) without DOP

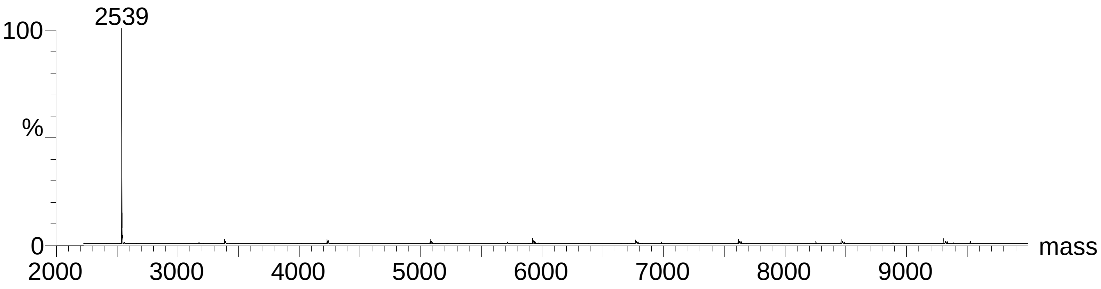

Ub-AMC with DOP

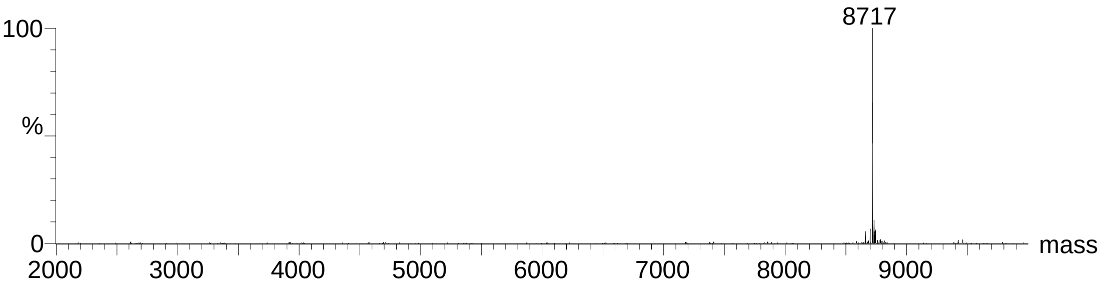

Ub-AMC without DOP

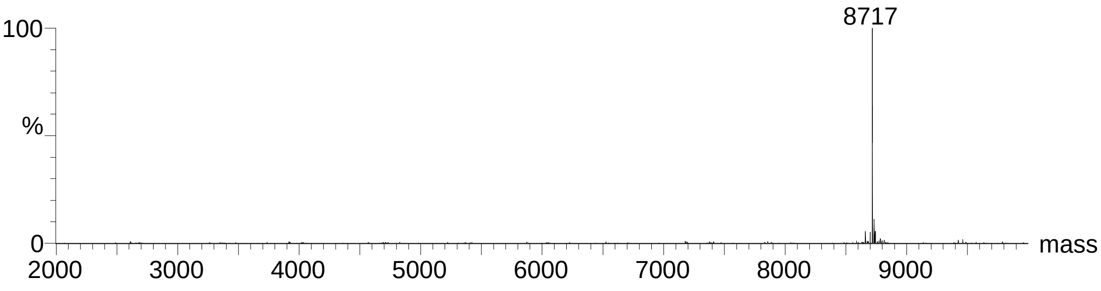

Figure S1 ESMS analysis of peptides **1a** – **c** and Ub-AMC after incubation in the Dop assay.

5. References

[1] Farid El Oualid, Remco Merkx, Reggy Ekkebus, Dharjath S. Hameed, Judith J. Smit, Annemieke de Jong, Henk Hilkmann, Titia K. Sixma and Huib Ovaa, *Angew. Chem. Int. Ed.*, **2010**, *49*, 10149 – 10153.

[2] Burns, K. E.; Cerda-Maira, F. A.; Wang, T.; Li, H.; Bishai, W. R.; Darwin, K. H., *Molecular Cell* **2010,** *39* (5), 821-827.

6. NMR spectra

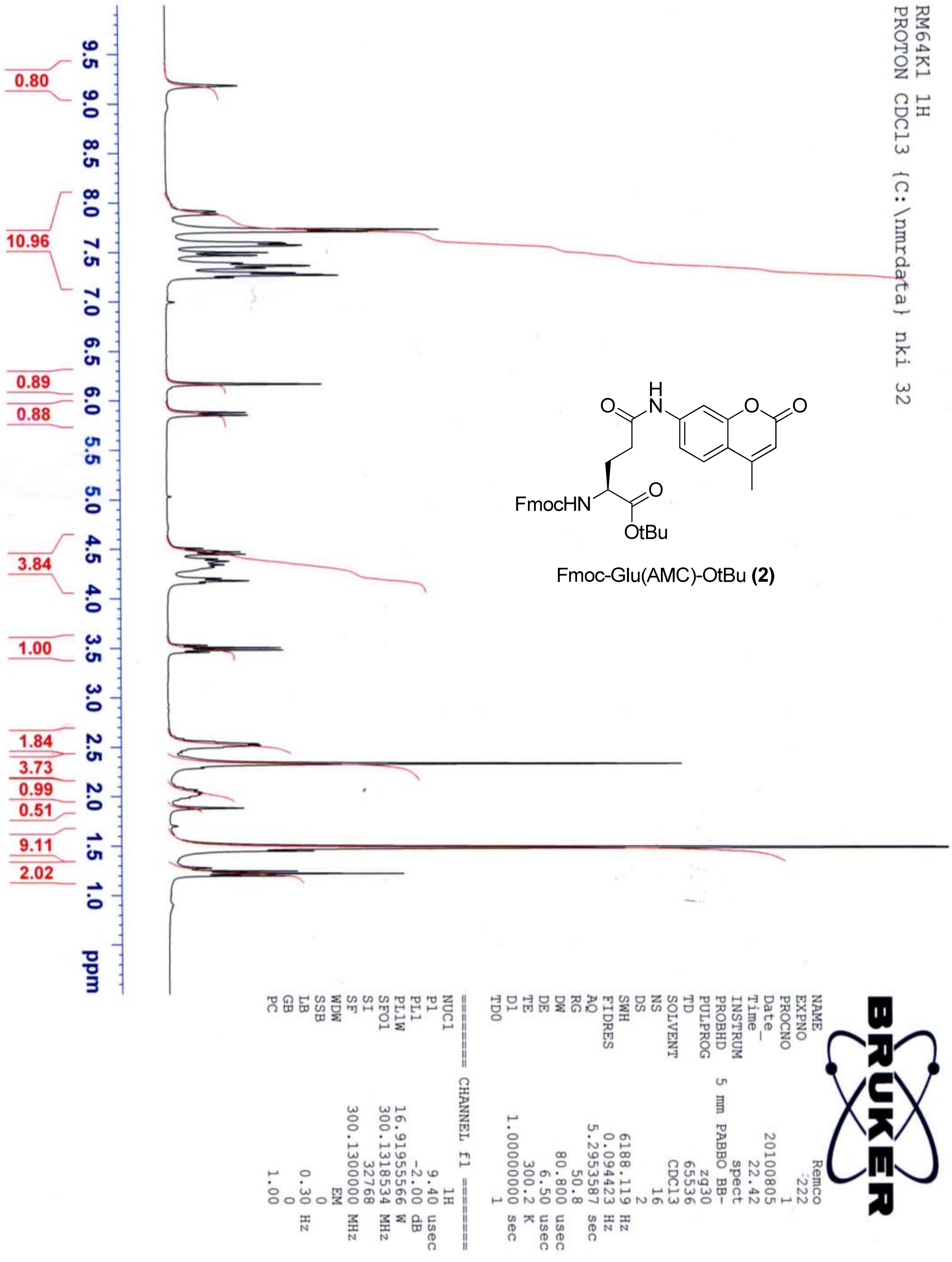

Fmoc-Glu(AMC)-OtBu **(2)**

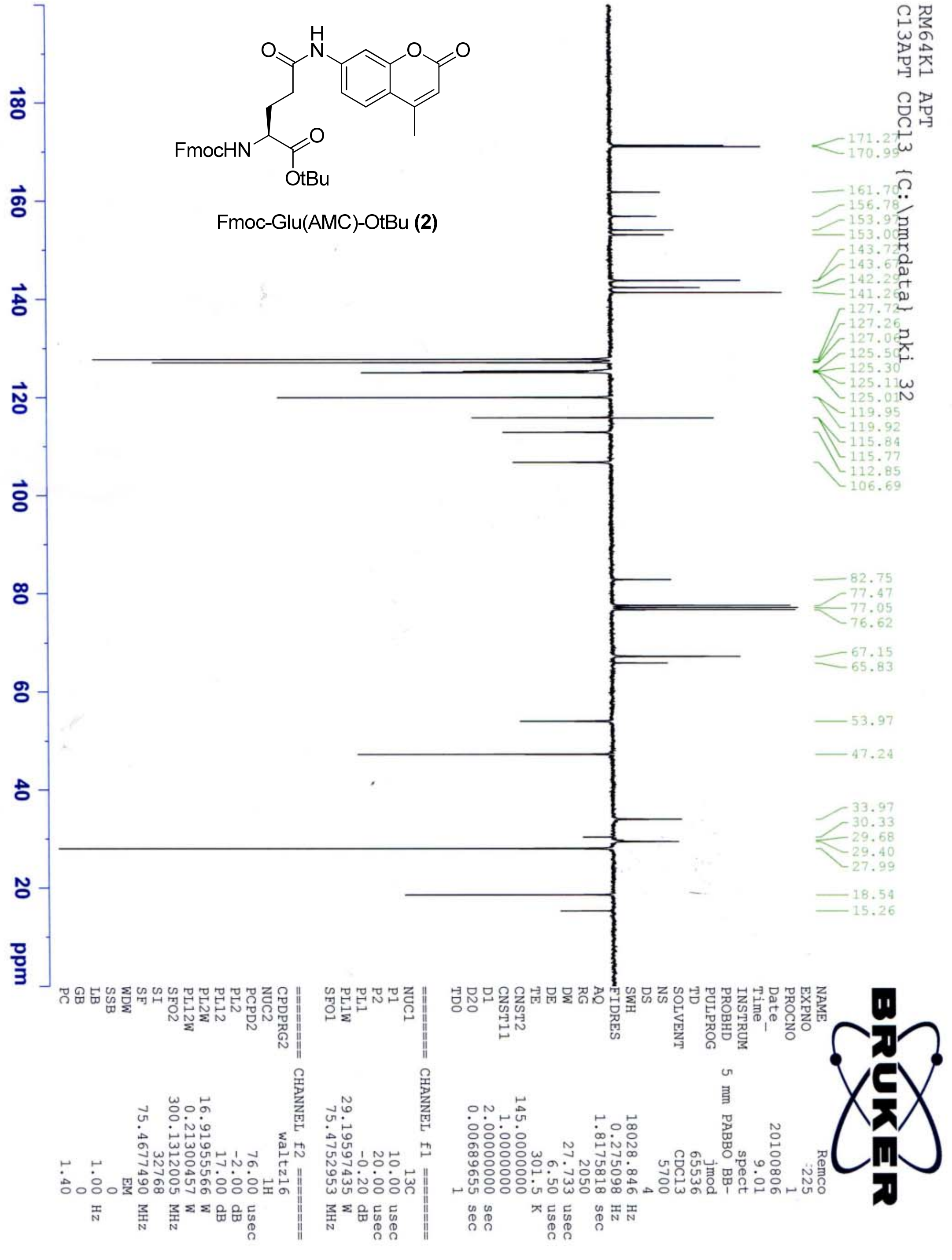
FmocHN
OtBu
Fmoc-Glu(AMC)-OtBu (2)
ppm

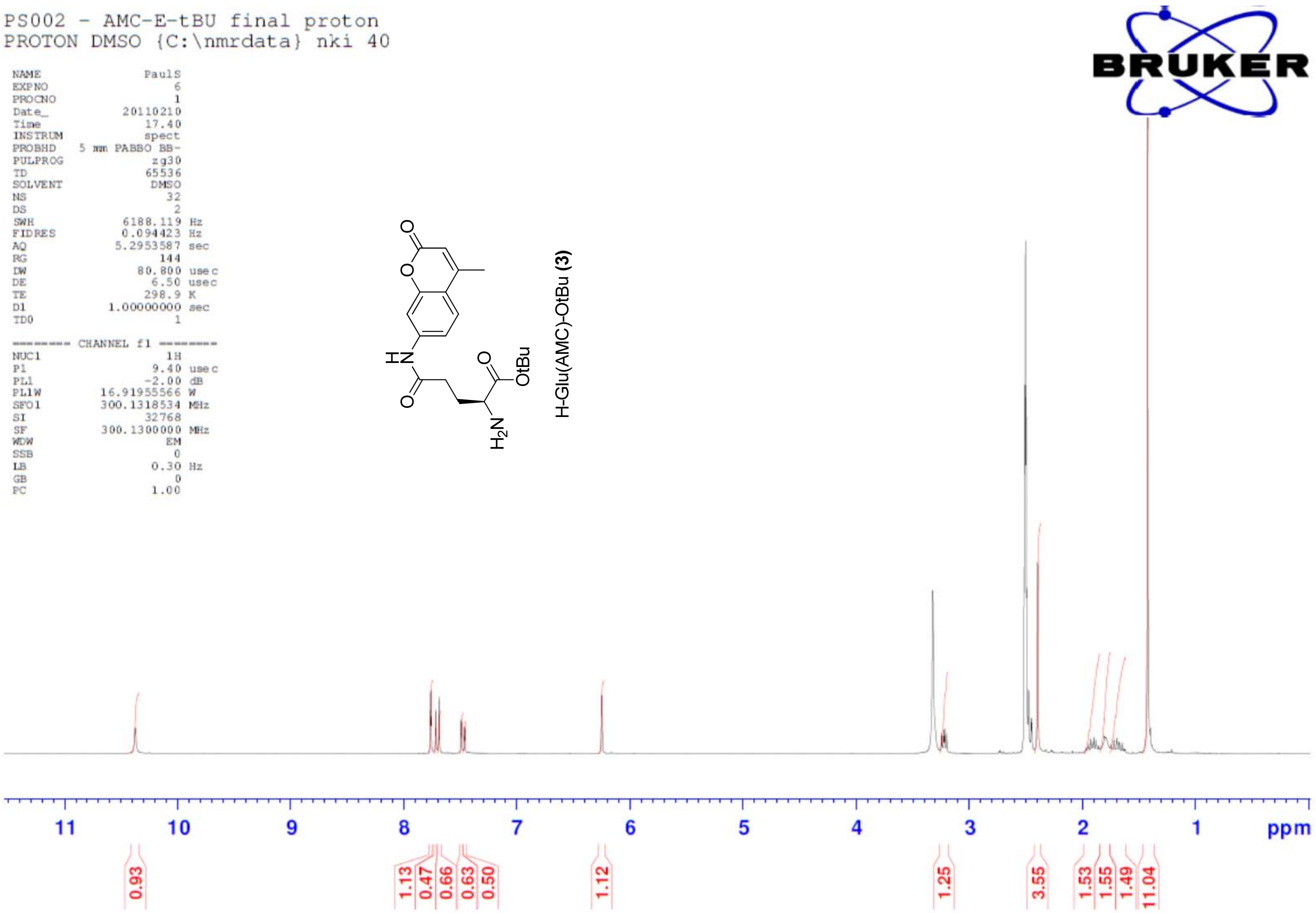

H-Glu(AMC)-OtBu **(3)**

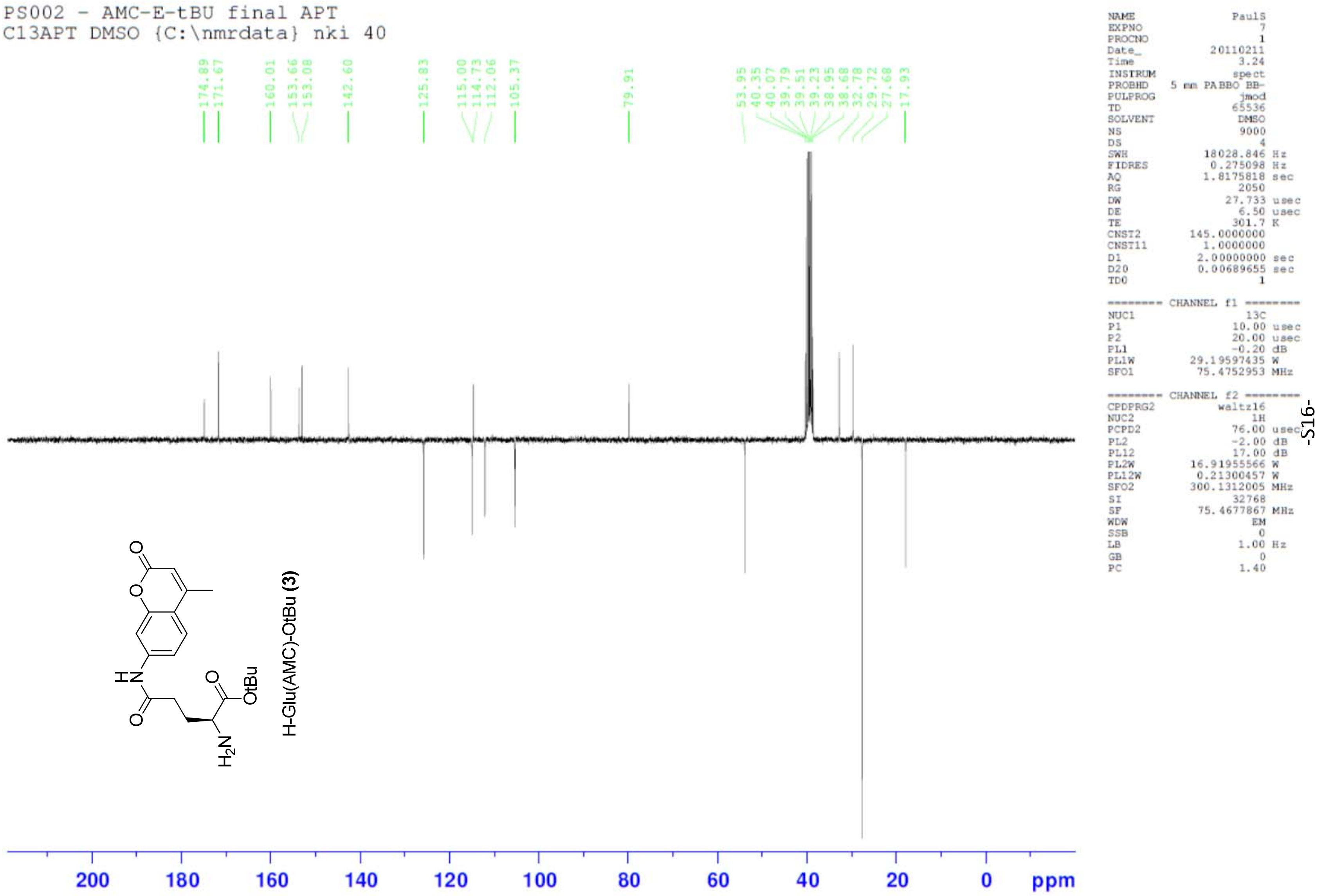

H-Glu(AMC)-OtBu **(3)**

7. HPLC-MS spectra

Pup(1-63)-Glu(AMC) (1a)

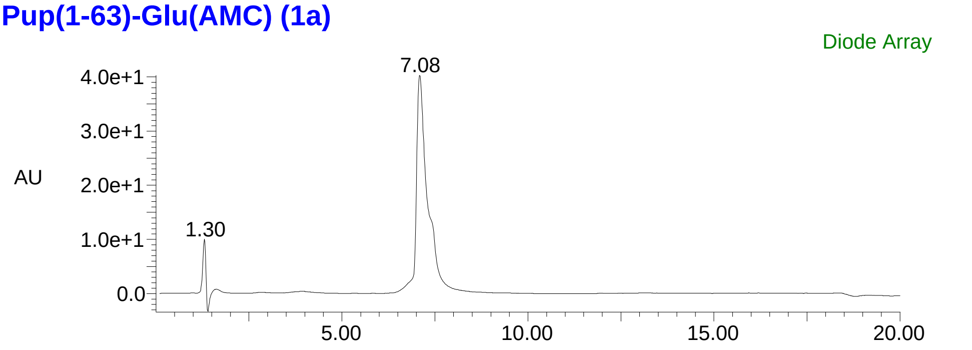

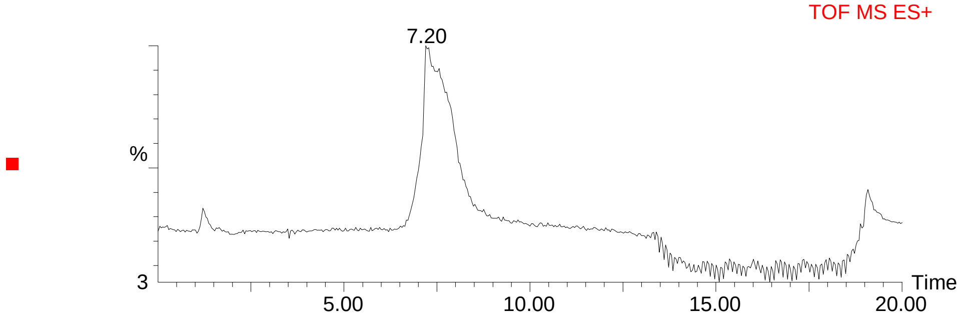

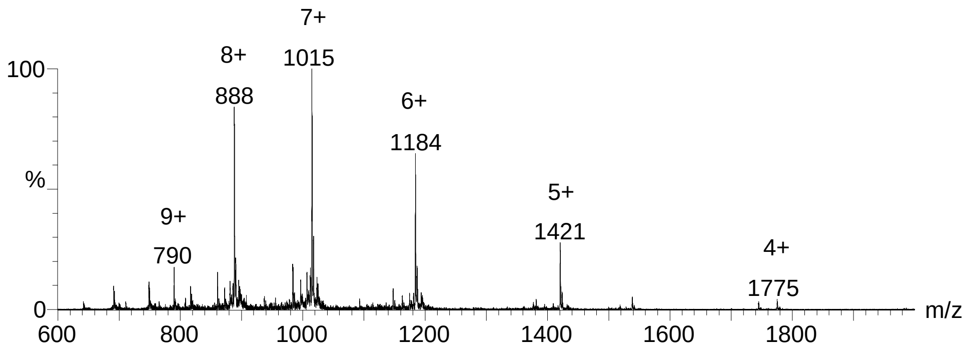

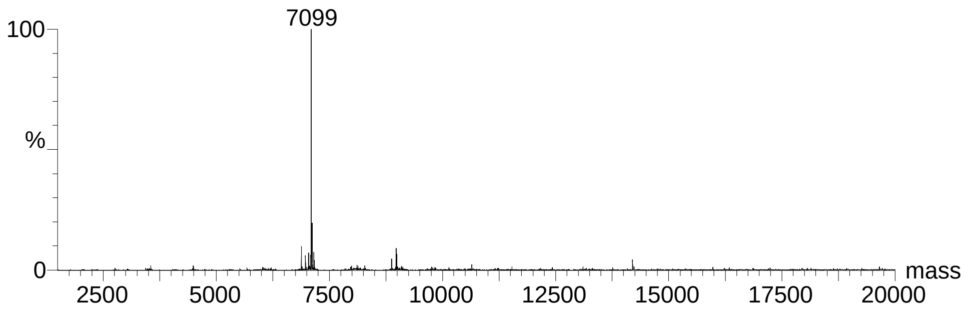

Pup(33-63)-Glu(AMC) (1b)

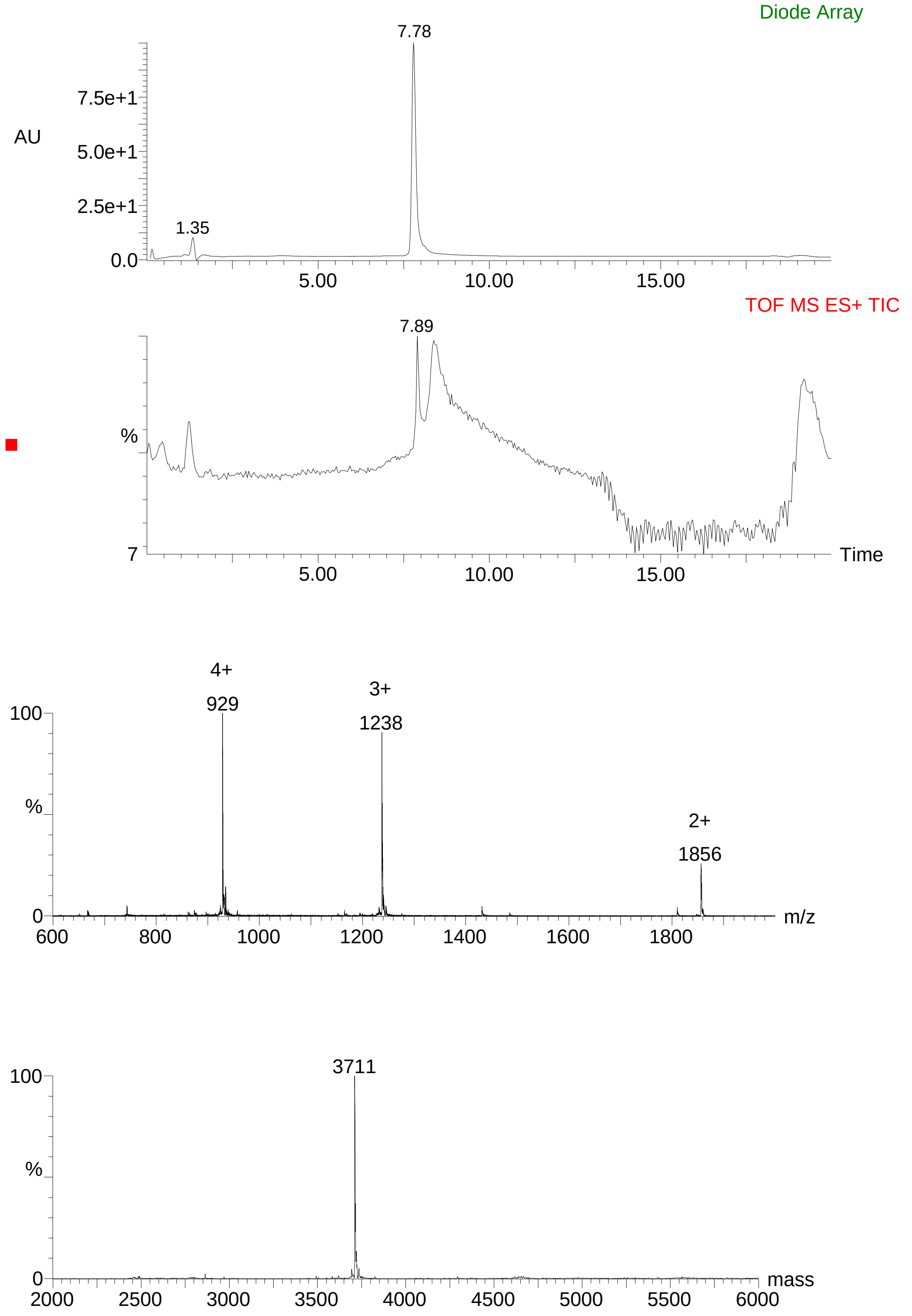

Pup(43-63)-Glu(AMC) (1c)

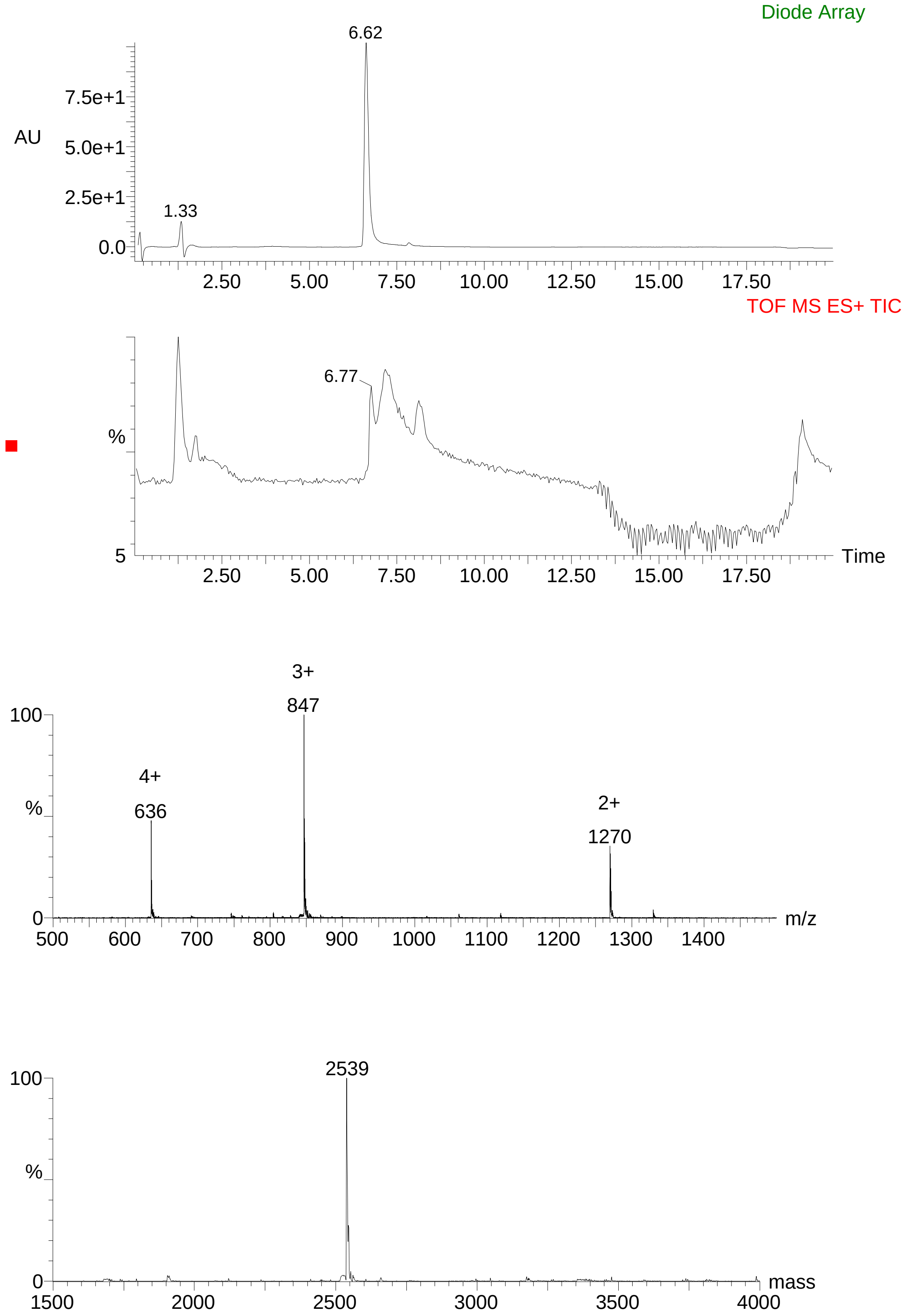

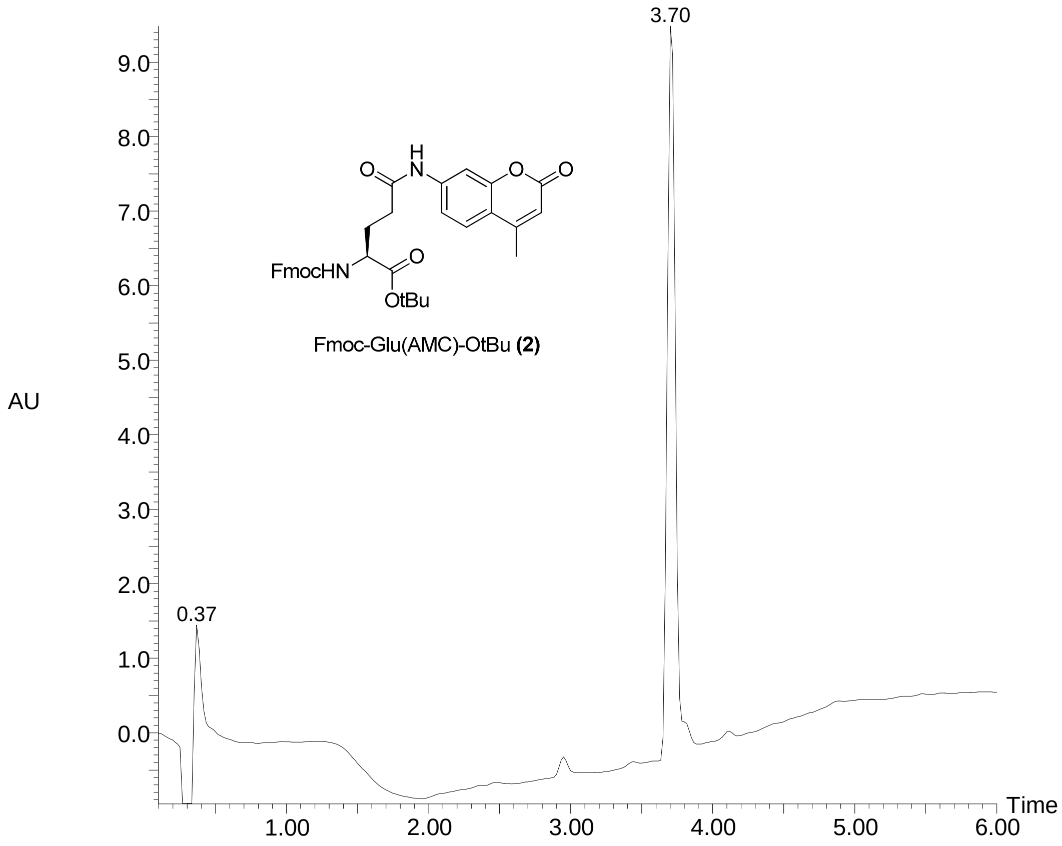
3.70
9.0
8.0
7.0
6.0
5.0
4.0
3.0
2.0
1.0
0.0
AU
0.37
FmocHN
OtBu
Fmoc-Glu(AMC)-OtBu **(2)**
Time
1.00
2.00
3.00
4.00
5.00
6.00

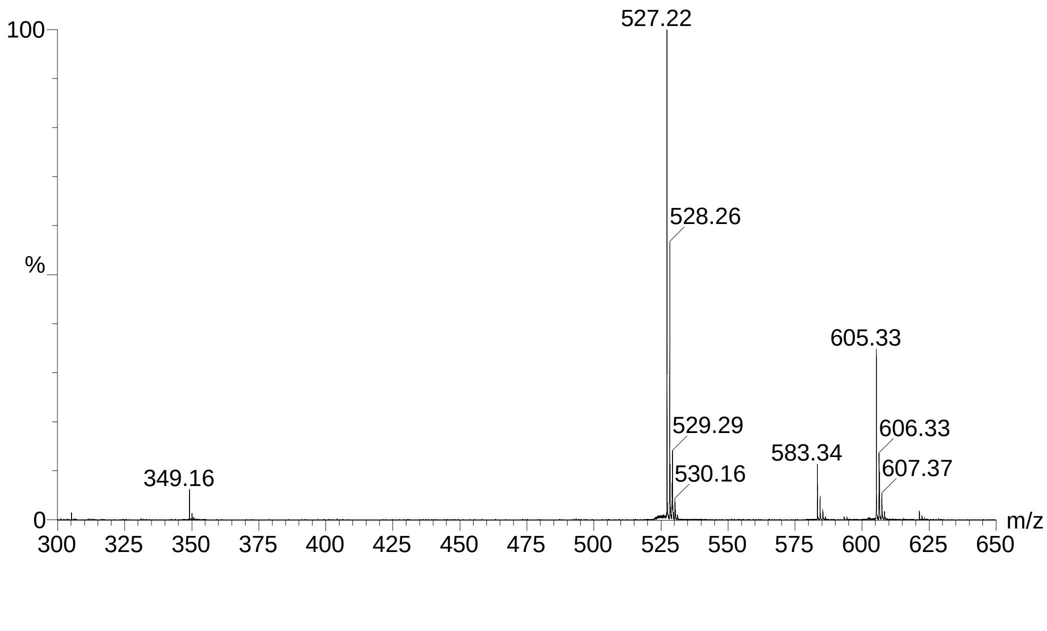
100
%
0
527.22
528.26
605.33
529.29
606.33
583.34
607.37
530.16
349.16
m/z
300
325
350
375
400
425
450
475
500
525
550
575
600
625
650

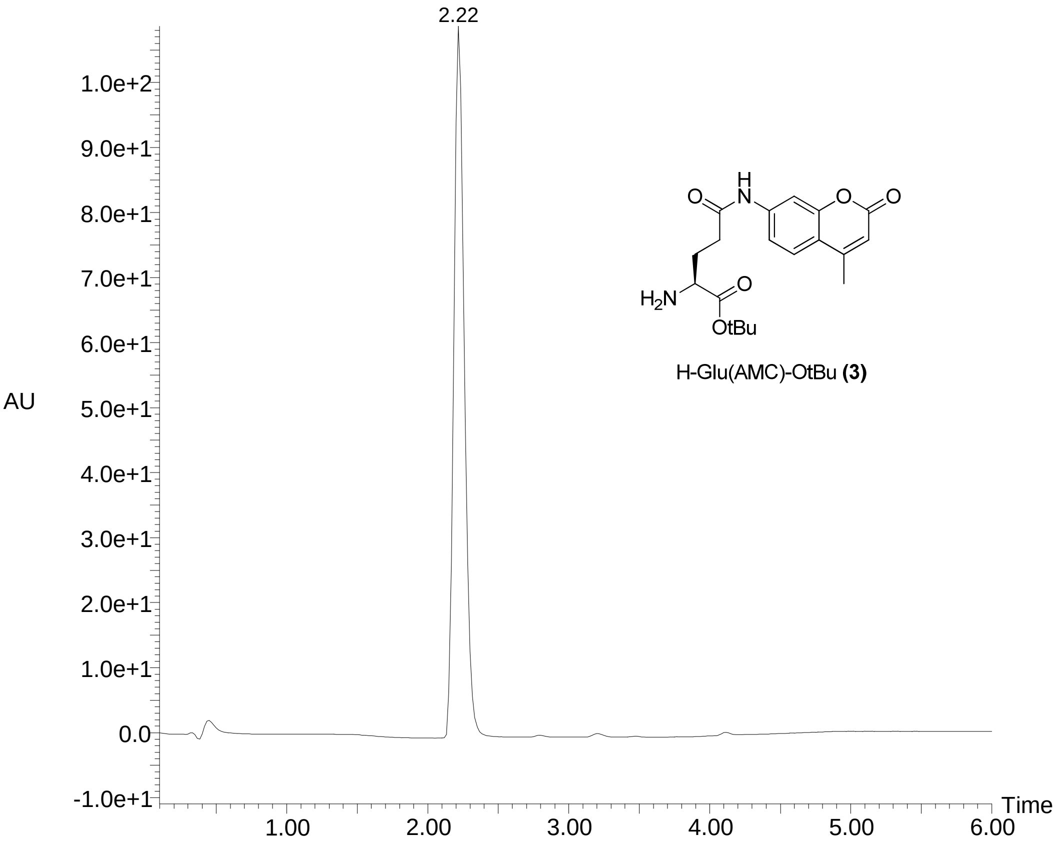

H-Glu(AMC)-OtBu **(3)**

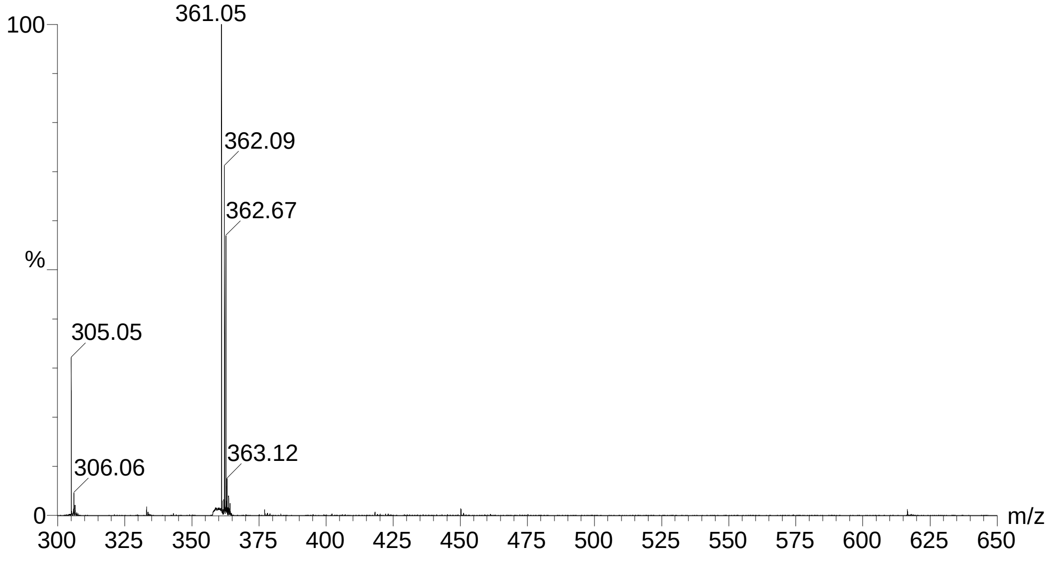

8. MS-MS analysis of compound 1a

A 2ul (10ug) aliquot of a 5ug/ul solution of **1a** in milliQ was digested with trypsin (1:40 enzyme to substrate ratio) in 50mM ammonium bicarbonate at 30°C overnight. The digest was then acidified with formic acid and an aliquot equivalent to 1ug was used for nLC-MS-MS analysis.

MSMS analysis of **1a** after tryptic digestion

Description	Score	Coverage	# Proteins	# Unique Peptides	# Peptides	# PSMs	Molecular Function	Cellular Component	Biological Process	Pfam IDs	# AAs	MW [kDa]	calc. pI	
RM-GluAMC	6820,47	100,00	1	12	12	129				Protein not found	64	6,9	4,16	
A2	Sequence	# PSMs	# Proteins	# Protein Groups	Protein Group Accessions	Modifications	ΔCn	IonScore	Exp Value	Charge	MH+ [Da]	ΔM [ppm]	RT [min]	# Missed Cleavages
High	LTEETDDLLDEIDDVLEENAEDFVR	21	1	1	RM-GluAMC		0,0000	129	1,20495E-13	2	2937,34477	2,55	31,27	0
High	GGGGGDDDDIAGSTAAGQER	31	1	1	RM-GluAMC		0,0000	118	1,61425E-12	2	1805,75749	2,28	15,39	0
High	RGGGGGDDDDIAGSTAAGQER	28	1	1	RM-GluAMC		0,0000	116	2,31191E-12	2	1961,85784	1,70	13,42	1
High	EKLTEETDDLLDEIDDVLEENAEDFVR	28	1	1	RM-GluAMC		0,0000	104	3,62218E-11	2	3194,48564	3,38	27,63	1
High	MAQEQTKR	2	1	1	RM-GluAMC		0,0000	57	1,93629E-06	2	991,50054	1,56	7,58	1
High	GGGGGDDDDIAGSTAAGQERREK	1	1	1	RM-GluAMC		0,0000	52	5,80724E-06	3	2218,99637	1,94	9,60	2
High	RGGGGGDDDDIAGSTAAGQERR	2	1	1	RM-GluAMC		0,0000	51	7,7085E-06	3	2117,95969	1,93	9,39	2
High	AYVQKGGe	1	1	1	RM-GluAMC	E8(AMC)	0,0000	51	7,76194E-06	2	1008,48076	2,19	13,31	1
High	MAQEQTK	6	1	1	RM-GluAMC		0,0000	46	2,47725E-05	2	835,39751	-0,43	11,64	0

MAQEQTKRGG GGGDDDDIAG STAAGQERRE KLTEETDDLL DEIDDVLEEN AEDFVRAYVQ KGG-Glu(AMC)

MSMS analysis of the C-terminal peptide segment after tryptic digestion

Peptide Summary

Sequence: AYVQKGGE, E8-AMC (157.05276 Da)
Charge: +2, Monoisotopic m/z: 504.74402 Da (+1.1 mmu/+2.19 ppm), MH+: 1008.48076 Da, RT: 13.31 min,
Identified with: Mascot (v1.27); IonScore:51, Exp Value:7.8E-006, Ions matched by search engine: 7/58
Fragment match tolerance used for search: 0.6 Da

Fragment Matches

Value Type: Theo. Mass [Da]

Ion Series | Neutral Losses | Precursor Ions

#1	b^+	b^{2+}	Seq.	y^+	y^{2+}	#2
1	72.04440	36.52584	A			8
2	235.10772	118.05750	Y	937.44144	469.22436	7
3	334.17614	167.59171	V	774.37812	387.69270	6
4	462.23472	231.62100	Q	675.30970	338.15849	5
5	590.32969	295.66848	K	547.25112	274.12920	4
6	647.35116	324.17922	G	419.15615	210.08171	3
7	704.37263	352.68995	G	362.13468	181.57098	2
8			E-AMC	305.11321	153.06024	1

Fragment Spectrum

Extracted from: D:\XL\Apr 12\120405\RM1.RAW #1253 RT: 13.31
ITMS, CID, z=+2, Mono m/z=504.74402 Da, MH+=1008.48076 Da, Match Tol.=0.6 Da

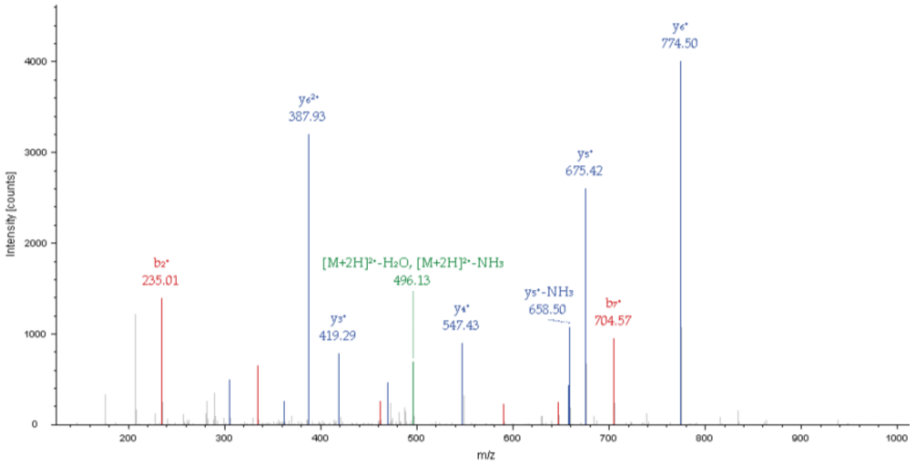

9. SDS-PAGE gel analysis of compounds 1a – 1c

NuPage 12% MES

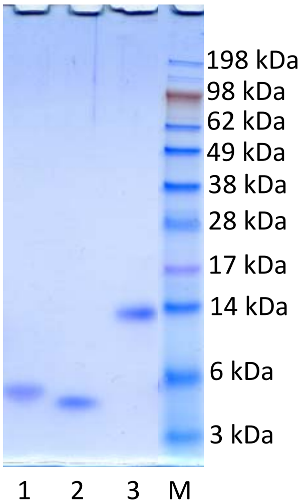

1 = **1b** (3.7 kDa)
2 = **1c** (2.5 kDa)
3 = **1a** (7.1 kDa)
M = Marker
